# A systematic review and meta-analysis of the hemodynamics and outcomes of the Myval balloon-expandable valve in patients with severe aortic stenosis and with aortic regurgitation

**DOI:** 10.1016/j.ijcha.2025.101641

**Published:** 2025-03-06

**Authors:** Elfatih A. Hasabo, Amira A. Aboali, Lina Hemmeda, Ammar Elgadi, Salma S. Alrawa, Alaa S. Ahmed, Malaz M. Abdalmotalib, Abdullatif Yasir H. Eissa, Mohammed Mahmmoud Fadelallah Eljack, Sherif Sultan, Osama Soliman

**Affiliations:** a Royal College of Surgeons in Ireland (RCSI) University of Medicine and Health Sciences, Dublin, Ireland; b Precision Cardiovascular Medicine & Innovation Institute (PCMI), Cardiovascular Research Institute Dublin (CVRI), Mater Private Network, Eccles Street, Dublin D07 KWR1, Ireland; c Damanhour Teaching Hospital, General Organization for Teaching Hospitals and Institutes, Damanhour, Egypt; d Faculty of Medicine, Alexandria University, Alexandria, Egypt; e Faculty of Medicine, University of Khartoum, Khartoum, Sudan; f Faculty of Medicine, University of Bakht Alruda, Al Dwuaym, Sudan; g Department of Vascular and Endovascular Surgery, Western Vascular Institute, University College Hospital, Galway, Ireland; h Euro Heart Foundation, the Netherlands

**Keywords:** Myval, Transcatheter aortic valve implantation, Aortic stenosis, Transcatheter valve-in-valve, Non-calcified aortic regurgitation

## Abstract

**Introduction:**

Transcatheter aortic valve implantation (TAVI) has been growing rapidly. We aim to evaluate the performance and outcomes of the Myval transcatheter heart valve (THV) in patients with severe aortic stenosis and its use in quantitative videodensimetry, transcatheter valve-in-valve (ViV), and non-calcified aortic regurgitation (NCAR).

**Methods:**

A systematic search was done in PubMed, Scopus, Web of Science, Embase, and Cochrane from inception to October 2024. We used the relevant keywords to include studies that reported the outcomes of patients with severe aortic stenosis who underwent TAVI using the Myval THV and off-label usage in transcatheter ViV and NCAR. Data analysis was done using R software.

**Results:**

A total of 29 studies were included in this study. The results of the mean aortic gradient at discharge, 30-day, one-year, and 2-year were 9.25 mmHg (95 % CI [8.20, 10.29]), 8.46 (95 % CI [7.57, 9.34]). 10.63 (95 % CI [9.12, 12.14]), and 7.2 (95 % CI [6.78, 7.63]), respectively. Additionally, the pooled percentages of patients with ≥ moderate aortic regurgitation were found in 1 % (95 % CI [1,2]) at discharge, 3 % (95 % CI [2,4]) at 30-day, 4 % (95 % CI [2,7]) at one-year follow-up and 5 % (95 % CI [3,8]) at 2-year. Furthermore, usage of the Myval THV in transcatheter ViV and NCAR led to a reduction in mean aortic gradient and incidence of aortic regurgitation, respectively.

**Conclusion:**

The Myval THV showed good safety and efficacy outcomes in short- and long-term follow-ups following the TAVI. Also, it showed promising results during transcatheter ViV and NCAR.

## Graphical abstract

**Figure:**
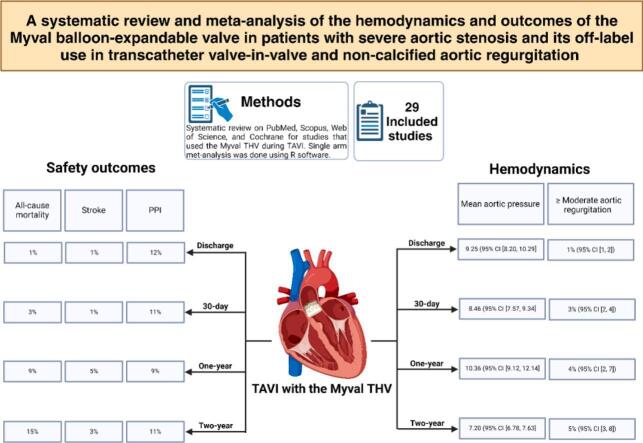

## 1 Introduction

Aortic stenosis is a common heart valve disorder, with clinical intervention typically required for severe symptomatic cases or severe asymptomatic cases associated with left ventricular systolic dysfunction (LVSD)[1]. It results from an inflammatory process triggered by endothelial damage caused by mechanical stress. This damage allows lipid infiltration, leading to fibrosis and thickening of the leaflets, which ultimately results in calcification[2]. The prevalence of aortic stenosis is significantly related to advanced age[1] and is likely the most frequent cause of sudden death among valvular heart diseases[3].

According to the National Echocardiography Database of Australia (NEDA), the incidence of aortic stenosis increased eightfold, from 5 cases per 1000 person-years in those under 30 to 40 cases per 1000 person-years in those over 80, with an overall incidence of about 18 cases per 1000 person-years[4]. Moreover, The percentage of people who died with severe aortic stenosis increased from 3.9 % among those older than 65 to 6.1 % among those older than 85[4].

Surgical aortic valve replacement (SAVR) has been the standard treatment for severe aortic stenosis. However, there has been a significant shift in recent years, with transcatheter aortic valve Implantation (TAVI) emerging as a highly effective alternative to surgery for an increasing number of patients with severe AS[5].

TAVI has been effectively performed in over 200,000 patients across 65 countries and is now regarded as the optimal approach for managing severe calcific aortic stenosis in patients with intermediate to high surgical risk scores[5]. Several TAVI systems have been approved and widely used, including SAPIEN 3, SAPIEN XT by Edwards Lifesciences, Lotus™ by Boston Scientific, and CoreValve®, Evolut™ PRO by Medtronic. However, specific reports have highlighted challenges during implantation or after the procedure in patients with low, intermediate, and high operative risks. These challenges include the need for a new permanent pacemaker (PPM), paravalvular leak (PVL), increased risk of valve dislocation, annular rupture, aortic regurgitation (AR), and the potential need for a second TAVI implantation[6], [7], [8], [9], [10].

The Myval, developed by Meril Lifesciences, is a balloon-expandable transcatheter heart valve (THV) with unique design features that make it easier for operators to use, thus improving delivery accuracy. Following the MyVal-1 study, the first in humans, Myval received approval from the Central Drugs Standard Control Organization (CDSCO) of India in October 2018 and obtained CE marking in the European Economic Area in April 2019[11]. By January 2023, the Myval THV has been approved for commercial use in 60 countries, with more than 8,000 TAVI procedures performed worldwide using this system[12].

Hemodynamics is essential for understanding heart health and function. An important hemodynamic parameter used to assess cardiac performance is the mean pressure gradient, which is mainly used to determine the severity of valve stenosis[13]. The Myval has significantly improved the mean pressure gradient across the valve, which helps restore normal blood flow and relieve symptoms associated with valve stenosis[14].

Moderate or severe AR after TAVI has been associated with higher short- and long-term mortality rates[15]. The MyVal-1 first-in-human trial demonstrated the safety and effectiveness of the Myval THV, showing low rates of AR[11]. Moreover, the quantitative videodensitometric aortography is considered an objective and precise method for assessing AR post-TAVI[16].

No previous *meta*-analysis has assessed multiple outcomes of TAVI using the Myval THV. Therefore, we aimed to conduct this *meta*-analysis to summarize the current evidence about the hemodynamic performance and outcomes of the Myval THV in patients with aortic valve diseases.

## 2 Methods and materials

This systematic review and *meta*-analysis assesses the efficacy and safety of the balloon expandable-valve Myval in patients with aortic stenosis and aortic disease. It is conducted in accordance with the Cochrane Handbook for systematic reviews of interventions[17], using the Preferred Reporting Items for Systematic Reviews and Meta-analysis (PRISMA) statement[18].

### 2.1 Search Strategy

We searched PubMed, Scopus, Web of Science (WoS), Cochrane, and Embase databases using the keywords; “Myval” OR “Myval Octacor” The specific search strategy that was run in each database is detailed in Supplementary Table 1***.*** All articles published until October 2024 were included for further screening against preset eligibility criteria.

**Table 1:** Summary of the included studies for patients used the Myval THV.

**Study**	**Study design**	**THV type**	**Population inclusion criteria underwent TAVI**	**Surgical risk status**	**Used expandable introducer sheath system during TAVI**	**Access site**	**Duration of follow-up**
Sharma et al. 2020 [11]	Prospective study (Single arm clinical trial)	Myval	Patients with severe symptomatic native AS selected for TAVI	Low	22 Fr or 24 Fr introducer sheath	Right common femoral artery 22 (73.3) Left common femoral artery 8 (26.7)	12 months
Kawashima et al. 2021 [20]	Retrospective study	Myval	Quantitative assessment of AR with videodensimtry	−	−	−	−
Ielasi et al. 2021 [21]	Case series	Myval	Trans-catheter valve-in-valve implantation with a novel BE device in patients with BHV failure	−	−	Trans-femoral venous access 5 (100 %)	−
Elkoumy et al. 2022 and 2023 [[22], [23]]	Retrospective study	Myval	Patients with severe BAV selected for TAVI	Low	Python 14-F introducer sheath 67 (98.5 %)Another introducer sheath (22-F) 1 (1.5 %)	Femoral access: 67 (98.5 %) patientOthers: 1 (1.5 %)	30 and 1 year
García-Gómez et al. 2022 [24]	Retrospective study	Myval	Patients with calcified severe AS who were treated with the implantation of the next generation BE Myval THV	Low	14Fr expandable introducer sheath	Transfemoral	30 days
Halim J et al. 2022 [25]	Prospective study	Myval	Patients with native symptomatic, severe AS	−	14 fr python sheath	Transfemoral (most), transapical	30 days & 1 year
Delgado-Arana JR, et al. 2022 [26]	Prospective study	Myval	Symptomatic patients with severe AS of the native valve	−	Sizes are 20 mm, 23 mm and 26 mm compatible with a 14fr expandable sheath (minimal vessel diameter 5.5 mm) and 29 mm compatible with 16fr expandable sheath (minimal vessel diameter 6 mm)	Transfemoral approach (206)	30 days
S. Santos-Martinez et al. 2022 [27]	Retrospective study	Myval	Symptomatic severe tricuspid AS	−	−	Transfemoral most common	−
Akyüz et al. 2022 [28]	Prospective study	Myval	Patients with AS in the medium–high-risk group	−	14-Fr expandable sheaths.	Transfemoral all	3 months
Abdelshafy et al. 2022 [29]	Retrospective study	Myval	−	−	−	−	−
Barki et al. 2022 [30]	Retrospective study	Myval	Patients with symptomatic, severe, native AS	Mostly are low-or intermediate-risk score,	−	Transfemoral (97 %)	30 days & 6 months
Elkoumy et al. 2023 [31]	Retrospective study	Myval Octacor	Patients with severe AS selected for TAVI	−	Expandable 14Fr Python introducer sheath 103 (100 %)	Transfemoral vascular access 103 (100 %)	
Sanchez-Luna et al. 2023 [32]	Prospective study (Single arm clinical trial)	Myval	Patients with symptomatic severe NCAR undergoing TAVR.	−	−	Transfemoral	30 days &12 months
Testa et al. 2023 [33]	Prospective study (Single arm clinical trial)	Myval	Patients with severe symptomatic AS	Mostly intermediate and high risk	14 fr python expandable sheath.	Transfemoral / 11 patients have been treated via transubclavian approach.	1 & 2 years
Moscarella et al. 2023 [34]	Prospective study	Myval	Patients with symptomatic, severe aortic BHV dysfunction undergoing transcatheter aortic ViV	−	14 Fr Python expandable sheath	Transfemoral	30 days & 1 year
Magyari et al. 2023 [35]	Retrospective study	Myval	Patients with significant AS	Low to high‐risk	14 fr python sheath	Tranfemoral (99 %), single trans subclavian implantation (1 %)	30 days & 1 year
Amat-Santos et al. 2023 [36]	Retrospective study	Myval	Patients with severe bicuspid AS selected for TAVI	Most had low or intermediate surgical risk,	14-Fr sheath		30 days
Halim, J et al. 2023 [37]	Retrospective study	Myval	Patients with symptomatic severe AS	−	A 14F sheath.	Transfemoral (90 %) transapical (10 %)	30 days
Holzamer et al. 2023 [38]	Retrospective study	Myval	Patients with AS and very large annular anatomy (mean area 765.5 mm2)	−	14F expandable Python sheath	Transfemoral	30 days
Boljevic et al. 2023 [39]	Prospective study	Myval	Patients underwent TAVI in Serbia	−	14 Fr Python introducer sheath	Transfemoral	
Moscarella et al. 2024 [40]	Retrospective study	Myval	Patients with symptomatic, severe, native AS	−	−	Transfemoral (97 %)	30 days, 1 year & 2 years
Magyari et al. 2024 [41]	Retrospective study	Myval	Patients with significant AS and BAV anatomy	Moderate to-high surgical risk	14 Fr Python sheath.	Transfemoral	30 days
			Patients with significant AS	Moderate to-high surgical risk	14 fr python sheath.	Transfemoral	30 days
Jose et al. 2024 [42]	Retrospective study	Myval Octacor	Severe symptomatic AS patients across 16 Indian centers who underwent TAVI with Myval Octacor THV	−	The expandable 14Fr Python introducer sheath	Transfemoral	30 days
Kilic et al. 2024 [43]	Prospective study (Single arm clinical trial)	Myval	Patients with severe AS	High or intermediate risk	−	Transfemoral (99 %)	30 days, 1 year & 2 year
Baumbach et al. 2024 (Landmark) [14]	RCT	Myval	Patients aged ≥ 18 years with symptomatic native AS	−	−	Transfemoral (99.7 %)	30 days
Ubben et al. 2024 [44]	Retrospective study	Myval	Patients with severe symptomatic AS	−	14 Fr Python sheath.	127 (95 %)	Discharge
Poletti et al. 2024 [45]	Prospective study	Myval	Patients with pure severe NAVR	High risk	−	Femoral 40 (98 %) Subclavian 1 (2.4 %)	30 days, 12 months, and latest available follow-up
Amber et al. 2024 [46]	Prospective study	Myval	Patients with severe symptomatic AS	High risk	14 Fr Python introducer sheath	Transfemoral 100 %	30 days

Abbreviation: AS; Aortic stenosis, TAVI; Transcatheter aortic valve implantation, BHV; Bioprosthetic Heart, BAV; bicuspid aortic valve, AR; aortic regurgitation, BE; balloon expandable, TAVR; Transcatheter aortic valve replacement, NCAR; non-calcified aortic regurgitation, NAVR; native aortic valve regurgitation, RCT; Randamized clinical trial, THV; transcatheter heart valve.

### 2.2 Eligibility criteria and study selection

In this systematic review, we included any original observational study, cohort study, retrospective study, or randomized control trial (RCT) that assessed the efficacy and/or safety of Myval in aortic stenosis or AR patients were deemed eligible for inclusion. Also, we excluded any case reports, non-human experimental studies, conference abstracts, and non-English articles from this systematic review.

### 2.3 Type of Intervention

All participants in the included studies were treated with the Myval or Myval Octacor THV.

### 2.4 Data extraction

Authors independently extracted the following data from all included studies:

1. **Baseline characteristics data**: Age, gender, aortic mean transvalvular gradient, aortic peak transvalvular gradient, body surface area (BSA), logistic Euroscore, Euroscore II, Society of Thoracic Surgery (STS) score, and risk factors.
2. **Summary of included studies**: Study design, THV type, population inclusion criteria underwent TAVI, expandable introducer sheath system during TAVI, Access site, Duration of follow-up
3. **Outcomes**: different outcomes were extracted and included the following:
• Device success, technical success, early safety, clinical efficacy.
• Hemodynamic outcomes: which included the left ventricular ejection fraction (LVEF), aortic valve mean gradient (mmHg), aortic valve peak gradient (mmHg), aortic valve area or effective orifice area (EOA) (cm^2^), aortic valve area indexed or indexed effective orifice area (iEOA) (cm^2^/m^2^).
• Aortic valve regurgitation
• The procedural outcomes
• The complications and safety outcomes
4. The AR using quantitative videodensitometry.
5. The off-label usage of the Myval THV in transcatheter valve-in-valve (ViV) and non-calcified aortic regurgitation (NCAR).

### 2.5 Quality Assessment

The Risk of Bias in Non-randomized Studies of Interventions (ROBINS-I) tool was used to assess the risk of bias in the included studies[19]. The assessment process was objective, considering A) pre-intervention domains, including confounding and selection bias; B) the intervention domain, including bias in the classification of interventions; C) the intervention domain, including bias due to deviations from intended interventions, bias due to missing data, bias in the measurement of outcomes and bias in the selection of the reported result. For each domain, the risk of bias is assessed as either low, moderate, critical, or severe, or no information is available. The overall risk of bias judgment for each study is the same for each domain, with the judgment of critical/ serious risk needing at least critical/ serious risk of bias in at least one domain.

### 2.6 Data Analysis

Data from the included studies were extracted and presented as calculated using the function “meta mean” presented as mean and 95 % CI for pooled continuous data and proportion and (95 % CI) for categorical data as forest plot and table. In the presence of heterogeneity, a random effect model was used during the *meta*-analysis. A subgroup *meta*-analysis was done according to the type of population used in this study.

## 3 Results

### 3.1 Literature search

**Fig. 1:**
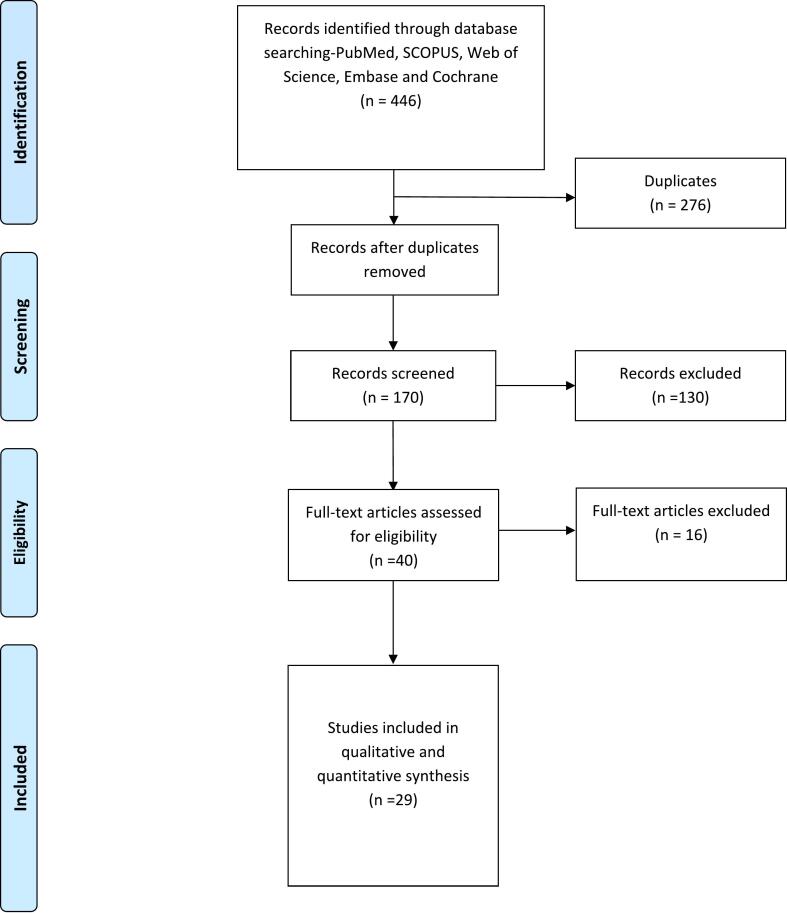
PRISMA Flow Diagram.

Following a search in medical databases for studies on the use of Myval in patients with aortic stenosis, we found 446 articles from PubMed, SCOPUS, Web of Science, Embase, and Cochrane. After removing duplicate studies, 259 articles remained for title and abstract screening. Of these, 40 full-text studies were assessed for eligibility and inclusion criteria. Finally, 29 studies met the inclusion criteria and were included in the systematic review[11], [14], [20], [21], [22], [23], [24], [25], [26], [27], [28], [29], [30], [31], [32], [33], [34], [35], [36], [37], [38], [39], [40], [41], [42], [43], [44], [45], [46]. Fig. 1 illustrates the study's search process and the flow of screening.

### 3.2 Summary and baseline clinical data of included studies

All studies included participants undergoing TAVI using Myval, most commonly through transfemoral access. Most studies followed patients for 30-day, while some followed them for one- or two-year. Each study provided details of the inclusion and exclusion criteria, as summarized in Table 1.

All included studies were predominantly conducted among elderly participants (i.e., aged 70 or older). However, the mean age varied between studies, with the youngest mean (69.9 ± 8.9) reported by Elkoumy et al., 2023, and the oldest mean (82 ± 6) reported by Moscarella et al., 2024. Most studies included patients with low or intermediate surgical risk, as indicated by the Society of Thoracic Surgery (STS), Logistic EuroSCORE, or EuroSCORE II. Hypertension was the most prevalent risk factor, which was reported in more than half of the participants. Other prevalent risk factors included diabetes mellitus and coronary artery disease. Details on the baseline characteristics of the patients are reported in Table 2.

**Table 2:** Baseline characteristic of patients using the Myval THV.

**Study**	**Total number**	**Age, years,**	**Male, %**	**Aortic mean transvalvular gradient, mmHg**	**Aortic peak transvalvular gradient, mmHg**	**Body surface area (BSA), m^2^**	**Logistic EuroSCORE**	**EuroSCORE II**	**Society of Thoracic Surgery (STS) score**	**Risk factors, number (%)**								
										**Hypertension**	**Prior atrial fibrilation**	**Diabetes mellitus**	**Peripheral vascular disease**	**Coronary artery diseases**	**Previous myocardial infarction**	**Previous revascularization**	**PCI**	**CABG**
Sharma et al. 2020 [11]	30	75.5 ± 6.7	22 (73.3 %)	47.4 ± 8.8	71.7 ± 13.0	−			6.4 ± 1.8				3 (10.0 %)	13 (43.3 %)	4 (13.3 %)		4 (13.3 %)	5 (16.7 %)
Kawashima et al. 2021 [20]	108	−	−	−	−	−	−	−	−	−	−	−	−	−	−	−	−	−
Ielasi et al. 2021 [21]	4	79.2 ± 5.1	0 (0 %)	−	−	−		12.4 ± 7.4	6 ± 3.3	5 (100 %)	3 (60 %)	1 (20 %)		3 (60 %)				
Elkoumy et al. 2022 [22]	68	72.6 ± 9.4	49 (72 %)	−	−	1.75 ± 0.24	−		3.54 ± 2.1	−	12 (18 %)	−	9 (13 %)	−	−	−	−	−
García-Gómez et al. 2022 [24]	100	80.0 ± 6.5	51 (51 %)	43 (37–––48)	70 (60–––79)	1.9 ± 0.2	−	2.2 ± 0.7	2.4 ± 0.8	74 (74 %)	12 (12 %)	30 (30 %)	8 (8 %)	39 (39 %)	−	−	−	2 (2 %)
Halim J et al. 2022 [25]	60	80.2 ± 6.6	30 (50 %)	37.1 ± 12.8	−	−	−	4.0 ± 2.8	−	45 (75 %)	23 (38 %)	26 (43 %)	11 (18 %)	32 (53 %)	−	−	−	7 (12 %)
Delgado-Arana JR, et al. 2022 [26]	130	80.9 ± 6.9	70 (53.9 %)	40 (33–47)	67 (53–77)	1.83 ± 0.22	−	−	3.2 (2.1–5.3)	−	38 (29.2 %)	44 (33.8 %)	11 (8.5 %)	52 (40 %)	−	−	−	7 (5.4 %)
S. Santos-Martinez et al. 2022 [27]	135	80.93 ± 6.80	72 (53.3 %)	41.71 ± 12.03	−	1.83 ± 0.22	−	3.65 ± 3.27	−	−	−	46 (34.1 %)	11 (8.1 %)	55 (40.7 %)	−	−	−	7 (5.2 %)
Akyüz et al. 2022 [28]	25	83 (75–87)	8 (32 %)	45.8 ± 9	77 ± 17	−	20.8 ± 12.8	−	5.4 ± 3.5	20 (80 %)	−	6 (24 %)	−	10 (40 %)	9 (36 %)	−	4 (16 %)	6 (24 %)
Abdelshafy et al. 2022 [29]	103	−	−	−	−	−	−	−	−	−	−	−	−	−	−	−	−	−
Barki et al. 2022 [30]	58	82 ± 6	29 (50 %)	43.3 ± 13.8	−	−	−	−	3.3 ± 1.8	52 (90 %)	18 (31 %)	12 (21 %)	18 (31 %)	35 (60 %)	8 (14 %)	−	25 (43 %)	3 (5 %)
Elkoumy et al. 2023 [31]	103	69.9 ± 8.9	66 (64 %)	47.5 (40––59.5)	77 (68–––94.7)	1.7 ± 0.2			3.5 (2.1––7.1)	59 (64.8 %)		52 (57 %)	3 (3.4 %)	48 (54 %)	5 (5.7 %)		8 (9.0 %)	12 (13.5 %)
Sanchez-Luna et al. 2023 [32]	113	78.4 ± 7.46	73 (64.6 %)	6.3 ± 3.5	−	−	−	3.48 ± 2.7	2.71 ± 1.7	97 (85.8 %)	35 (31.0 %)	30 (26.5 %)	11 (9.7 %)	−	6 (5.3 %)	−	6 (5.3 %)	4 (3.5 %)
Testa et al. 2023 [33]	100	80.7 ± 7.7	62 (62 %)	41.7 ± 12.0	−	1.8 ± 0.2	−	−	6.3 ± 3.3	72 (72 %)	30 (30 %)	23 (23 %)	9 (9 %)	22 (22 %)	8 (8 %)	−	11 (11 %)	5 (5 %)
Moscarella et al. 2023 [34]	33	71.6 ± 14.9	23 (69.7 %)	37.4 ± 15.5	58.2 ± 28.9	1.8 ± 0.3	19.0 ± 12.8	9.6 ± 6.1	4.0 ± 2.6	25 (75.8 %)	8 (24.2 %)	7 (21.2 %)	6 (18.2 %)	8 (24.2 %)	4 (12.1 %)	−	6 (18.2 %)	6 (18.2 %)
Magyari et al. 2023 [35]	100	74.7 ± 7.2	63 (63 %)	48.4 ± 14.6	82.3 ± 24.3	1.94 ± 0.2	15.7 ± 15.5	4.8 ± 4.9	5.6 ± 3.9	95 (95 %)	18 (18 %)	40 (40 %)	10 (10 %)	−	24 (24 %)	−	39 (39 %)	22 (22 %)
Amat-Santos et al. 2023 [36]	122	73.0 ± 8.2	95 (77.8 %)	52.3 ± 14.2	81.7 ± 22.6	1.8 ± 0.2			4 (2.1–5.1)		19 (15.6 %)		12 (9.8 %)	42 (34.4 %)				9 (7.4 %)
Halim, J et al. 2023 [37]	120	80.2 ± 6.3	64 (53 %)	37.4 ± 13.5	−	−	−	4.0 ± 2.8	−	85 (71 %)	39 (33 %)	43 (36 %)	17 (14 %)	56 (47 %)	−	−	−	13 (11 %)
Holzamer et al. 2023 [38]	10	76.1 ± 7.0	10 (100 %)	46.4 ± 9.8	−	2.04 ± 0.31	−	7.63 ± 13.00	2.66 ± 1.37	−	−	−	−	−	−	−	−	−
Boljevic et al. 2023 [39]	13	72 ± 13	7 (53 %)	48 ± 48	−		mean of 7.17	−	mean of 2.72	−	−	−	−	6 (46.1 %)	4 (30.8 %)	−	1 (7.7 %)	4 (30.8 %)
Moscarella et al. 2024 [40]	58	−	−	−	−	−	−	−	−	−	−	−	−	−	−	−	−	−
Magyari et al. 2024 [41]	Bicuspid 52	71 ± 7.1	34 (65.4 %)	47.6 ± 15.9	79.9 ± 24.1	1.92 ± 0.23	12.2 ± 10.4	3.3 ± 3.2	5.2 ± 3.3	51 (98.1 %)	7 (13.5 %)	18 (34,6%)	9 (17.3 %)	−	9 (17.3 %)	−	12 (23.1 %)	4 (7.7 %)
	Triuspid 217	76 ± 6.9	131 (60.4 %)	47.8 ± 15.5	80.2 ± 25.7	1.95 ± 0.24	15.5 ± 15.2	5.2 ± 5.4	6.4 ± 4.4	211 (97.2 %)	46 (21,2%)	98 (45,2%)	31 (14.3 %)	−	53 (24.4 %)	−	79 (36.4 %)	42 (19.4)
Jose et al. 2024 [42]	123	70.07 ± 8.33	77 (62.6 %)	54.31 ± 18.19	−	1.70 ± 0.18	−	−	3.20 (1.80–5.05)	75 (60.9 %)	−	60 (48.8 %)	4 (3.3 %)	53 (43.1 %)	6 (4.9 %)	−	−	11 (8.94 %)
Kilic et al. 2024 [43]	207	80.7 ± 6.6	94 (45 %)	43.4 ± 18	−	1.8 ± 0.2	14.5 ± 7.4	5.2 ± 2.4	4.01 ± 1.9	171 (83 %)	−	57 (28 %)	−	107 (52 %)	−	−	60 (29 %)	20 (10 %)
Baumbach et al. 2024 (Landmark) [14]	384	80.0 ± 5.7	191 (49.7 %)	39.9 ± 14.0	65.2 ± 21.6	−	−	−	2.6 (1.7–4.0)	256 (66.7 %)	94 (24.5 %)	111 (28.9 %)	−	55 (14.3 %)	26 (6.8 %)	−	30 (7.8 %)	13 (3.4 %)
Ubben et al. 2024 [44]	134	81.0 ± 5.9	89 (66.4 %)	42 ± 14	69 ± 22	−	16 ± 12	4.7 ± 4.8	4.7 ± 6.1	123 (92 %)	−	34 (25 %)	−	−	−	−	59 (44 %)	19 (14 %)
Poletti et al. 2024 [45]	41	79 (76––84)	30 (73.1 %)	5 (3–7)	−	−	−	−	2.56 (1.93–3.60)	36 (88 %)	16 (39 %)	13 (32 %)	4 (9.8 %)	−	2 (4.9 %)	−	2 (4.9 %)	−
Amber et al. 2024 [46]	100	73.8 ± 6.5	50 (50.0 %)	46.8 ± 6.6	−	−	−	−	4.1 ± 1.6	67 (67.0 %)	−	77 (77.0 %)	−	43 (43.0 %)	−	−	−	−

Data are presented as: mean ± SD or median (IQR).

Abbreviation: PCI; Percutaneous coronary intervention, CABG; Coronary artery bypass graft.

### 3.3 Quality assessment of included studies

The quality assessment was done using the ROBINS-I tool, which showed a low risk of bias among the included studies. Further details about the quality assessment are found in Supplementary Table 2.

### 3.4 Hemodynamic outcomes and aortic regurgitation of Myval THV at discharge following TAVI

**Fig. 2:**
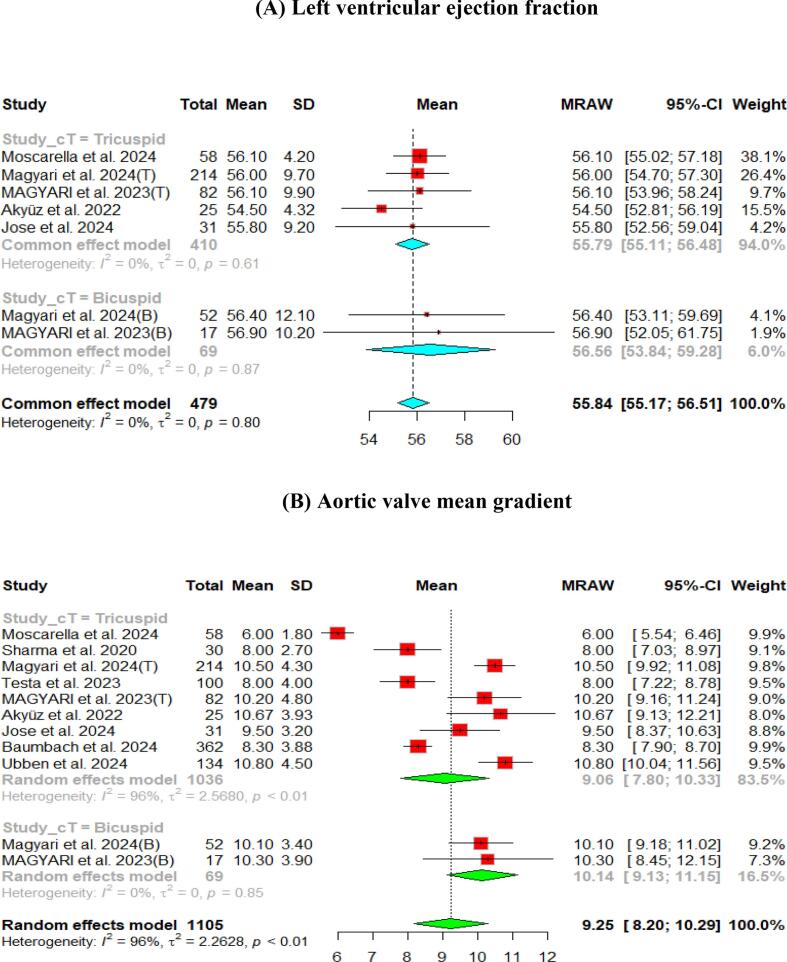

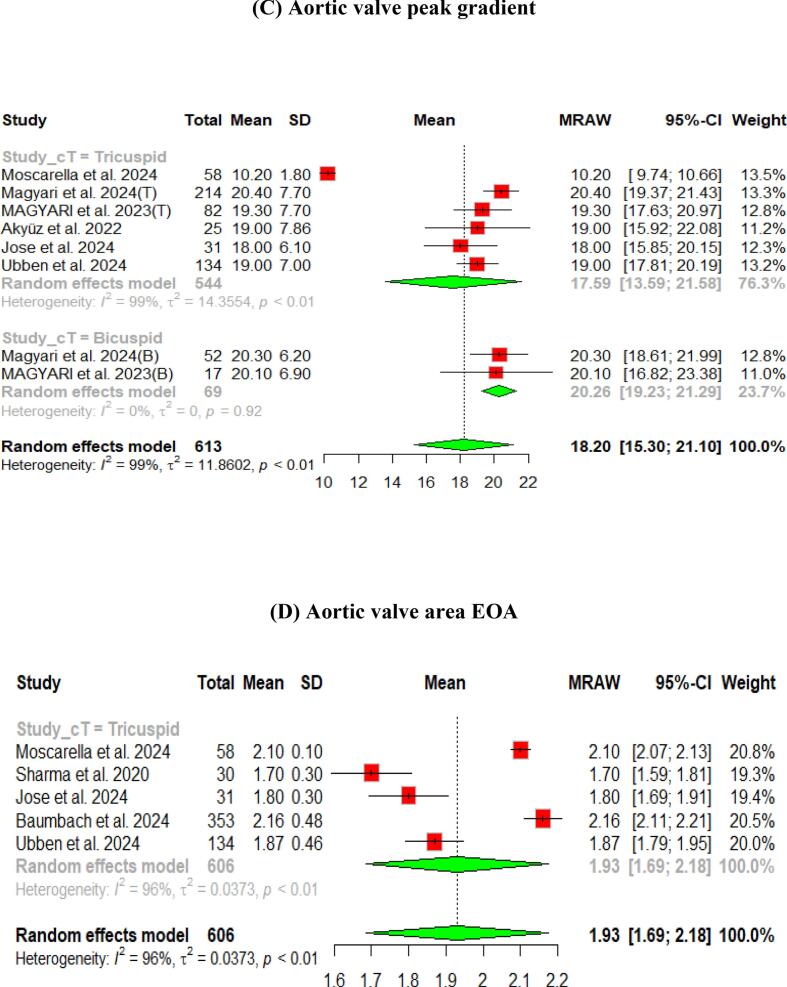

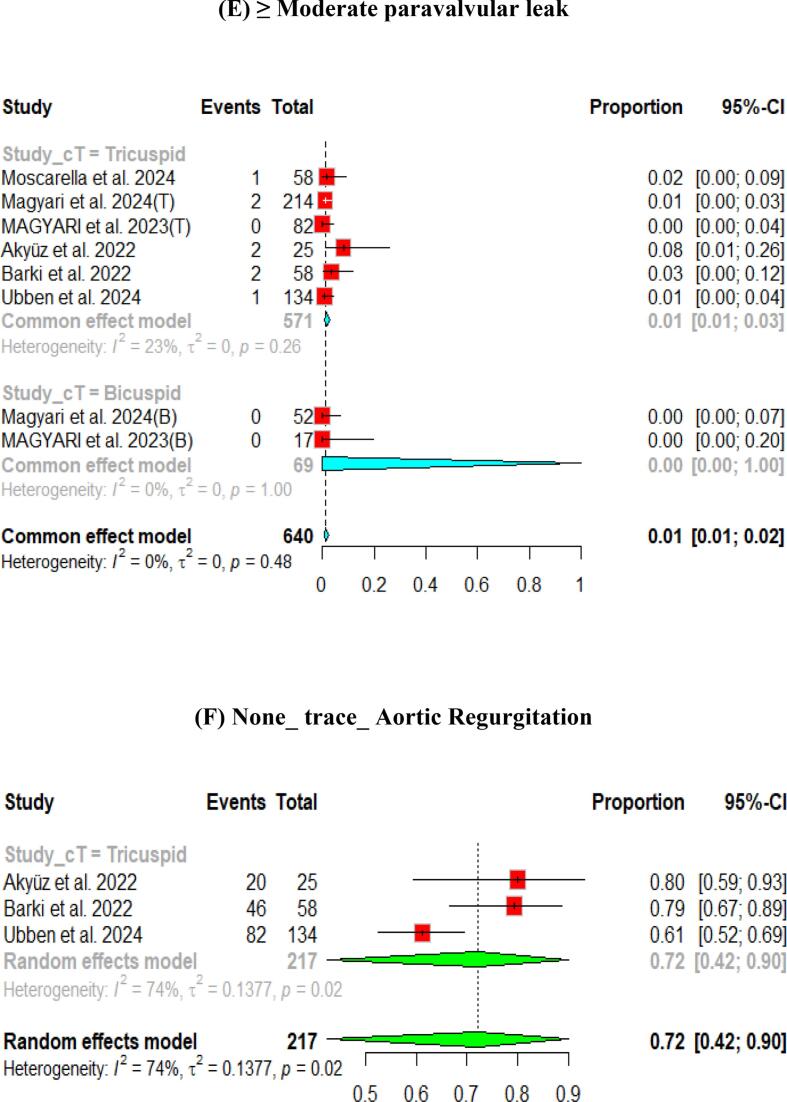

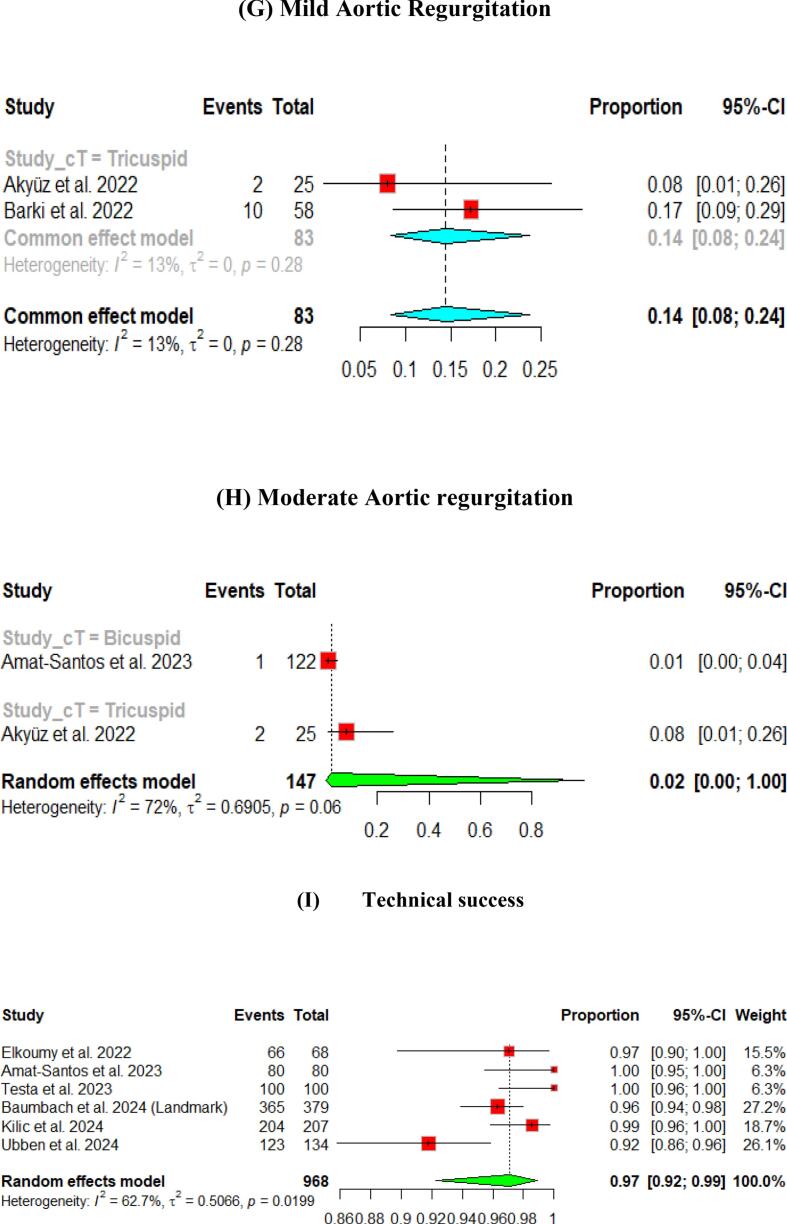
Hemodynamic outcomes of Myval THV in patients with severe aortic stenosis at post discharge: (A) Left ventricular ejection fraction Fraction, (B)Aortic valve mean gradient, (C) Aortic valve peak gradient, (D) Aortic valve area EOA, (E) Moderate paravalvular leak, (F) None/trace aortic regurgitation, (G) Mild Aortic Regurgitation, (H) Moderate Aortic regurgitation. (I) Technical success.

The hemodynamic outcomes were assessed after TAVI at multiple visits. The pooled mean LVEF at discharge was found to be 55.84 % (95 % CI [55.17, 56.51]), and the results were similar for patients with the bicuspid and tricuspid aortic valve (55.79 % vs 56.56 %, respectively) (Fig. 2). Furthermore, the pooled results showed that the mean aortic valve pressure gradient is 9.25 mmHg (95 % CI [8.20, 10.29]). While in the bicuspid valve population, the pooled mean aortic valve pressure gradient was 10.14 mmHg (95 % CI [9.13, 11.15]), and the tricuspid valve population had a mean of 9.06 mmHg (95 % CI [7.80, 10.33]) for the mean aortic valve pressure gradient. Following TAVI, the pooled effective orifice area was 1.93 cm^2^ (95 % CI [1.69, 2.18]). Additional detailed information about hemodynamics is shown in Fig. 2.

Aortic regurgitation and its severity were assessed across the studies. A paravalvular leak of ≥ moderate was observed in 1 % (95 % CI [1], [2]) of the pooled patients, and details of the severity of AR are shown in Fig. 2.

### 3.5 Hemodynamic outcomes and aortic regurgitation of the Myval THV at 30-day following TAVI

**Fig. 3:**
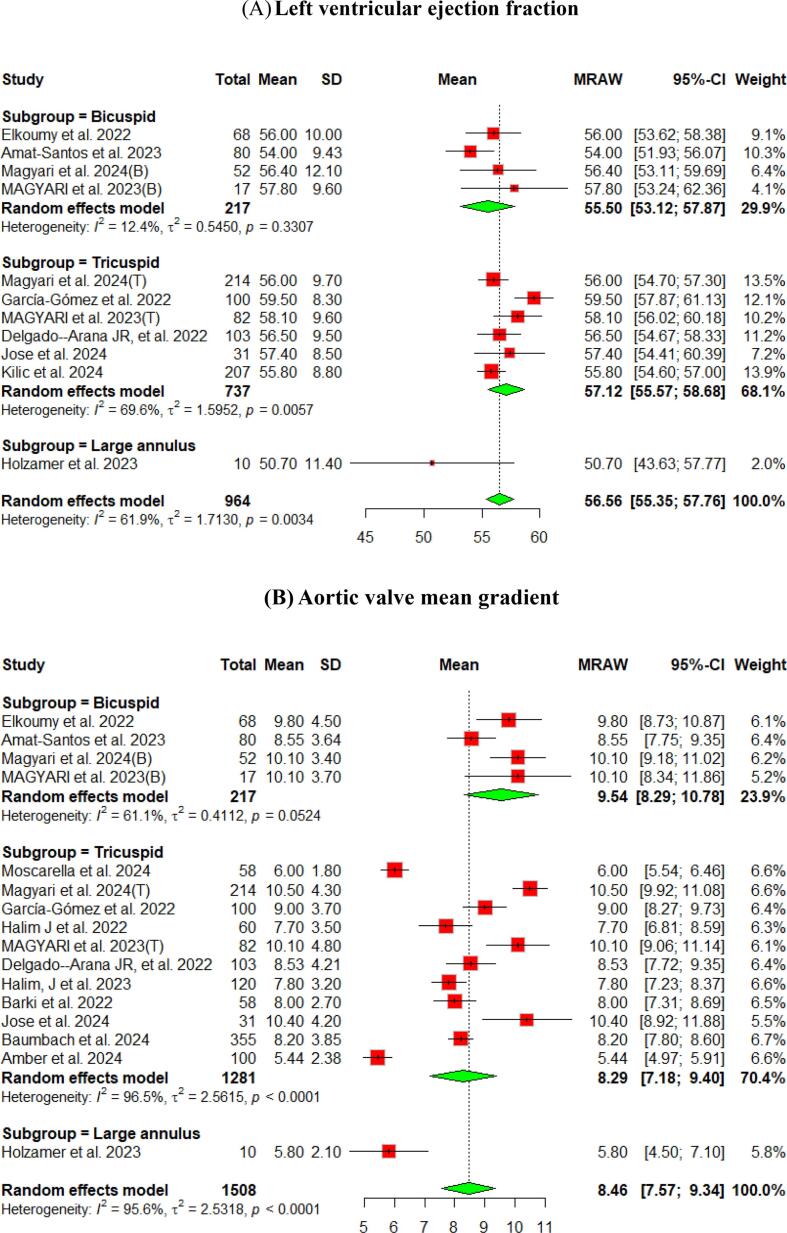

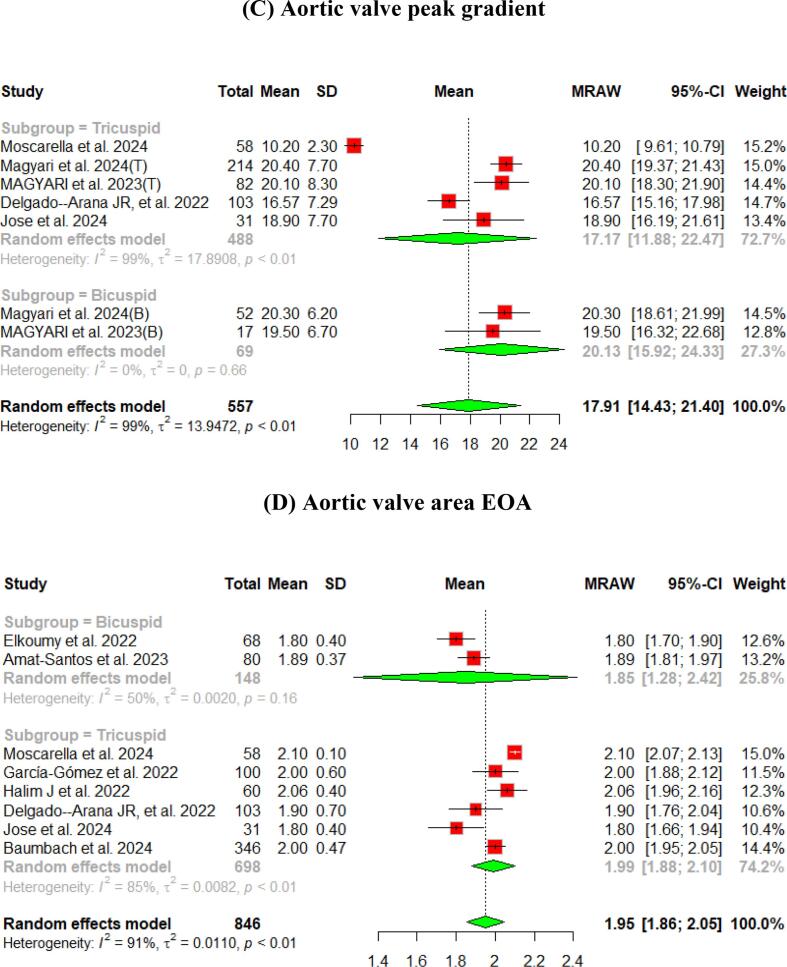

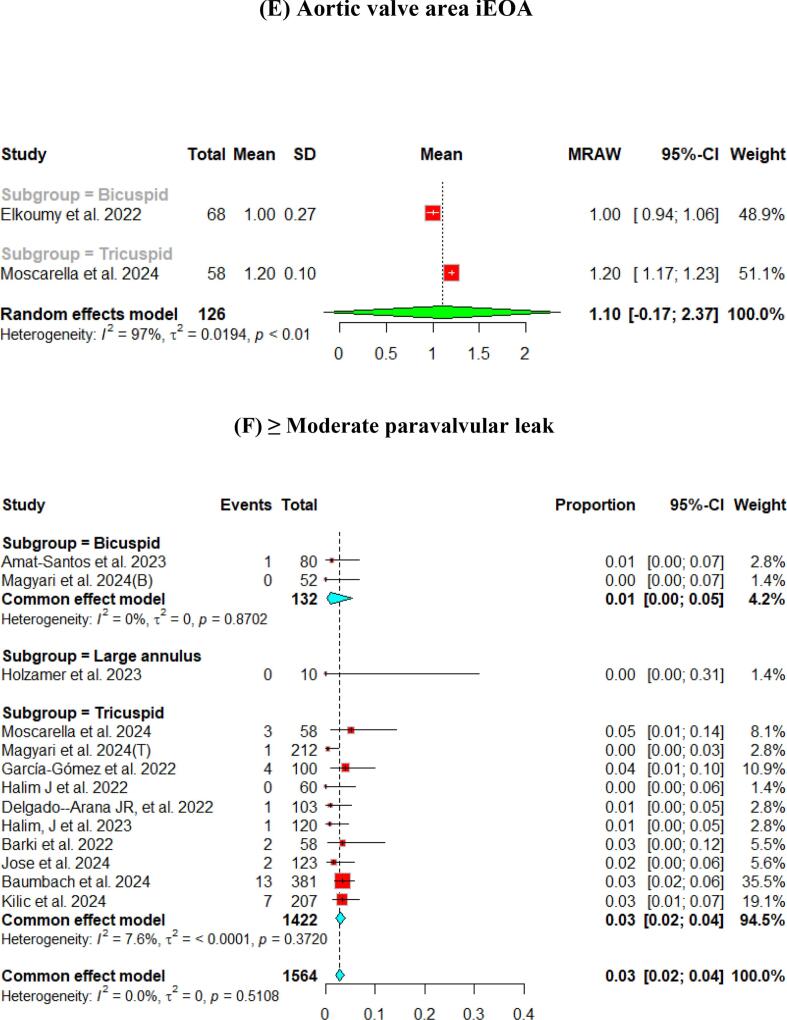

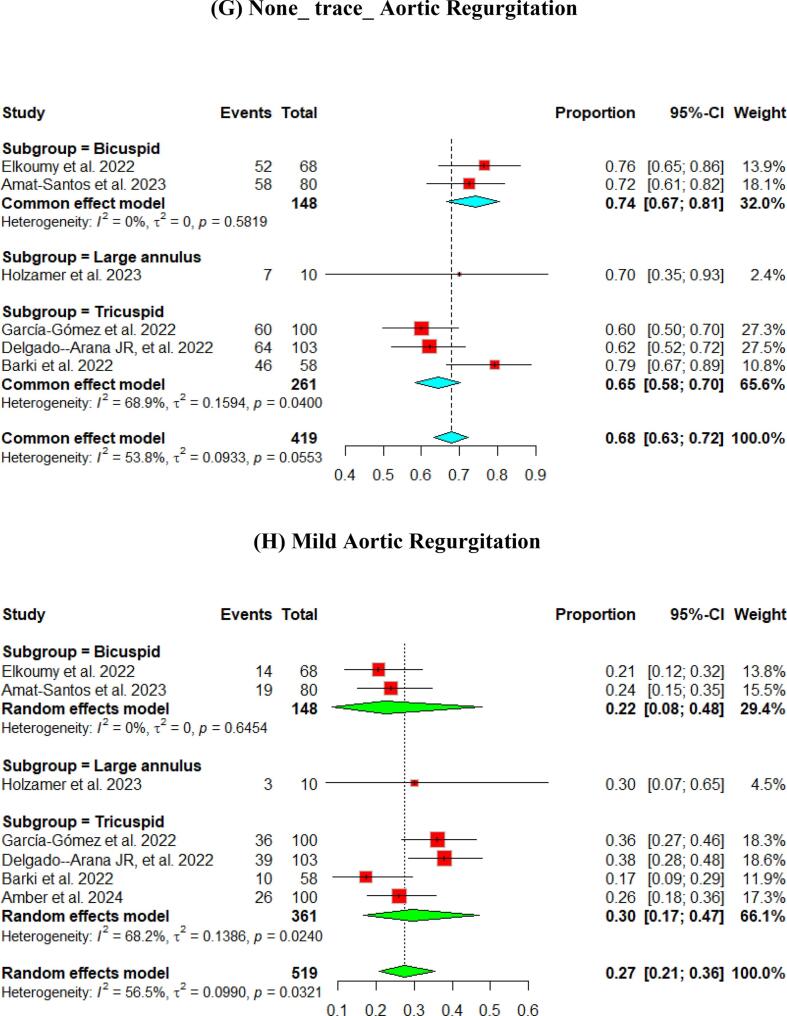

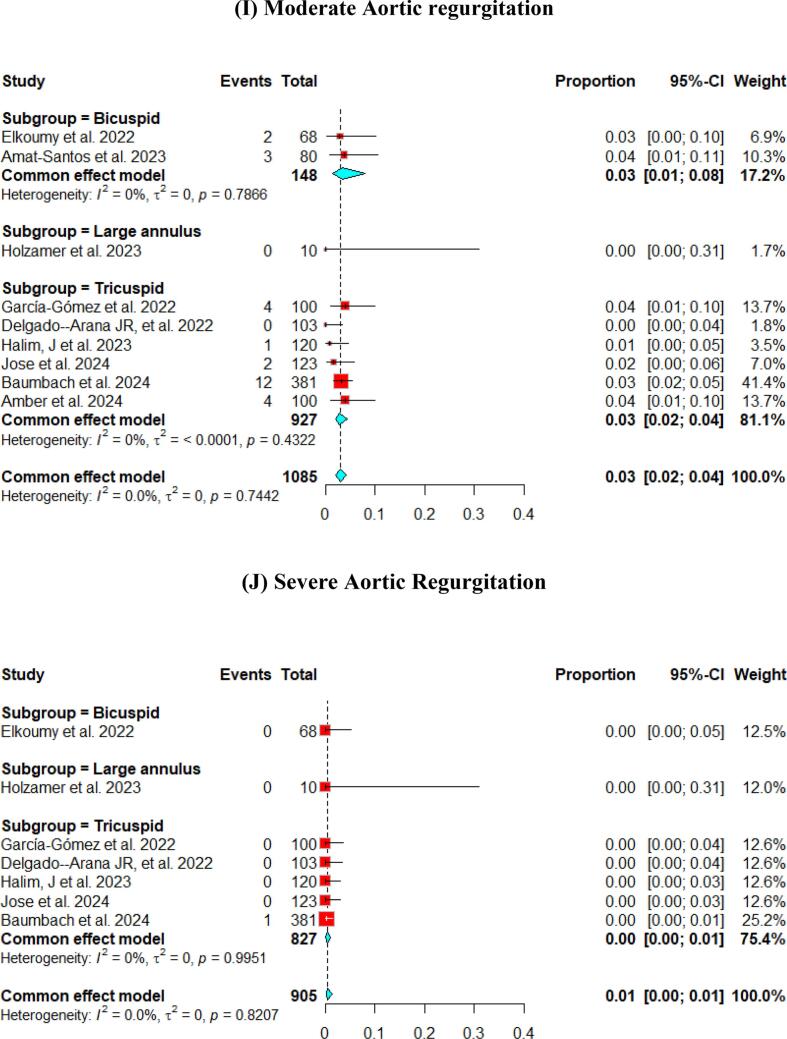

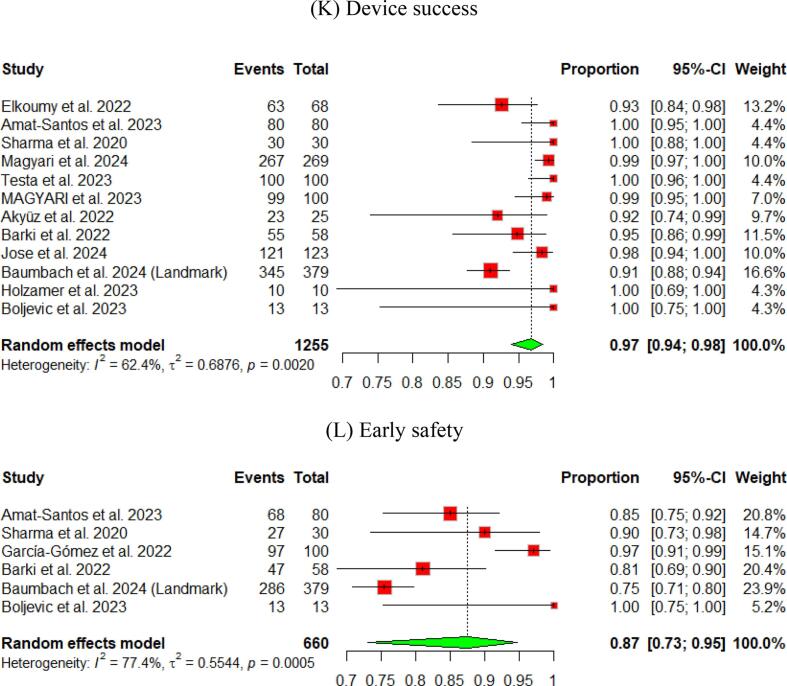
Hemodynamic outcomes of Myval THV in patients with severe aortic stenosis at 30-day: (A)Left ventricular ejection fraction, (B) Aortic valve mean gradient, (C) Aortic valve peak gradient, (D) Aortic valve area EOA, (E) Aortic valve area iEOA (F) Moderate paravalvular leak, (G) None/trace Aortic Regurgitation, (H) Mild Aortic Regurgitation, (I) Moderate Aortic regurgitation, (J) severe Aortic Regurgitation, (K) Device success, (L) Early safety.

At the 30-day follow-up. The pooled LVEF slightly increased from the baseline visit, reaching 56.65 % (95 % CI [55.53, 57.76]). It was higher in the tricuspid population (57.12 %) compared to the bicuspid population (55.50 %) (Fig. 3). The pooled mean aortic valve pressure gradient was 8.46 (95 % CI [7.57, 9.34]). The effective orifice area at the 30-day follow-up showed similar results to those at discharge, which was 1.93 cm2 (95 % CI [1.69, 2.18]). Other details are shown in Fig. 3.

Assessment of AR showed that only 3 % (95 % CI [2 %, 4 %]) of patients experienced ≥ moderate paravalvular leak. Fig. 3 depicts further details about the severity of AR.

### 3.6 Hemodynamic outcomes and aortic regurgitation of Myval THV at one- and two-year following TAVI

**Fig. 4:**
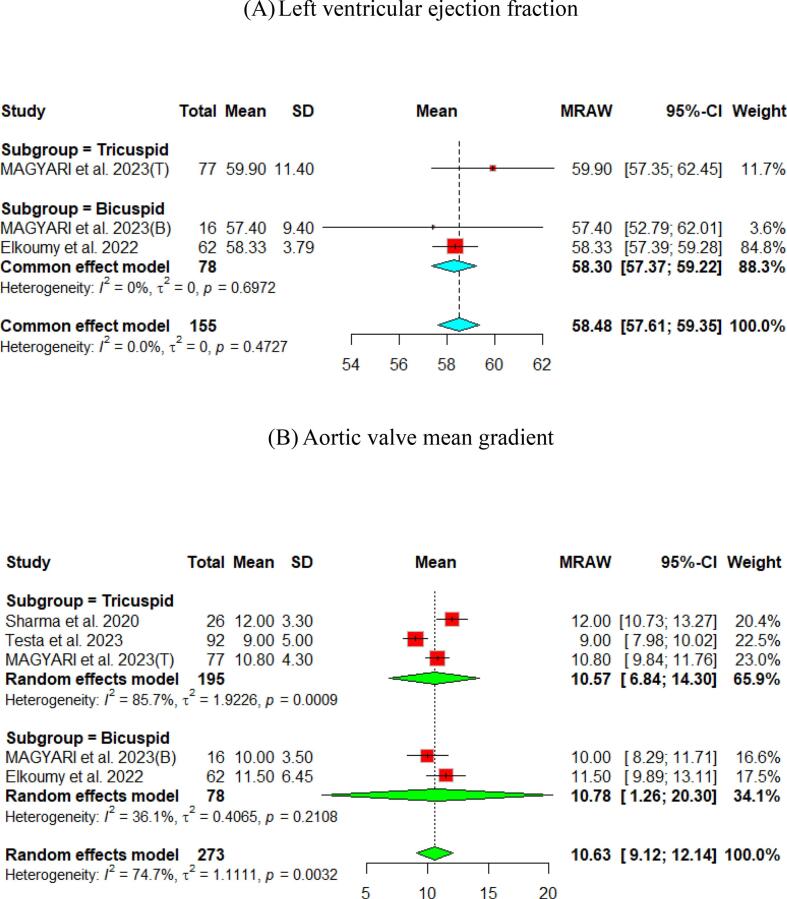

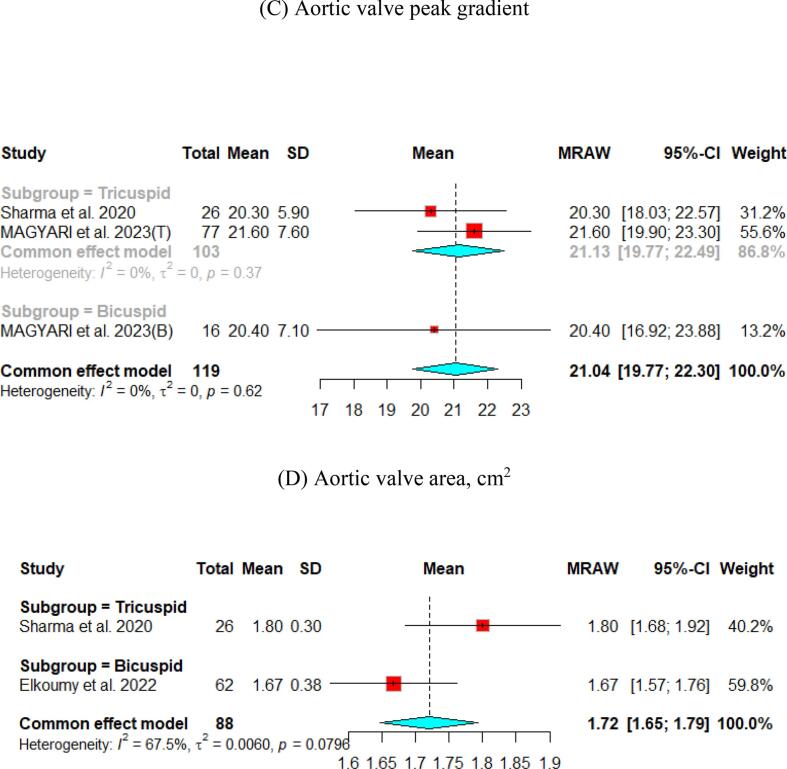

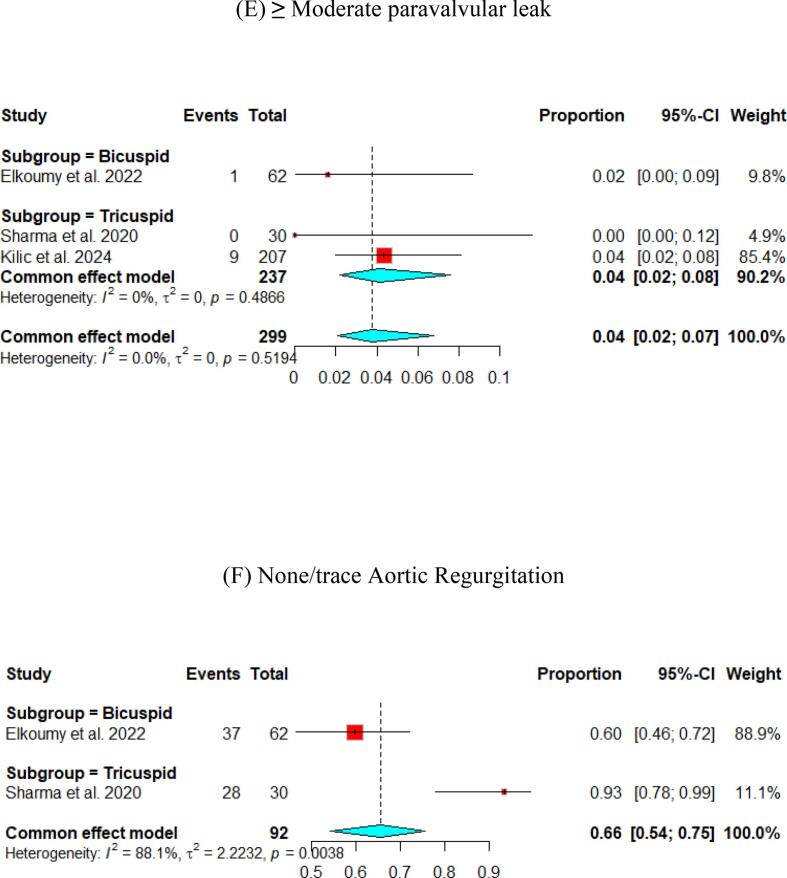

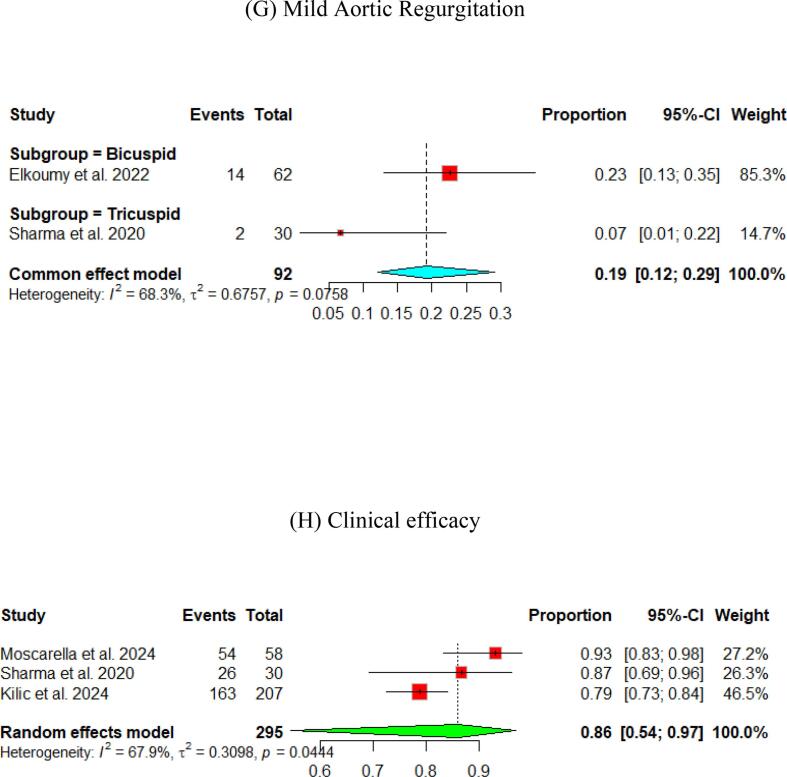
Hemodynamic outcomes of Myval THV in patients with severe aortic stenosis at 1 year: (A) Left ventricular ejection fraction Fraction, (B)Aortic valve mean gradient, (C) Aortic valve peak gradient, (D) Aortic valve area (E) ≥ Moderate paravalvular leak, (F) None/trace Aortic Regurgitation, (G) Mild Aortic Regurgitation, (H) Clinical efficacy.

**Fig. 5:**
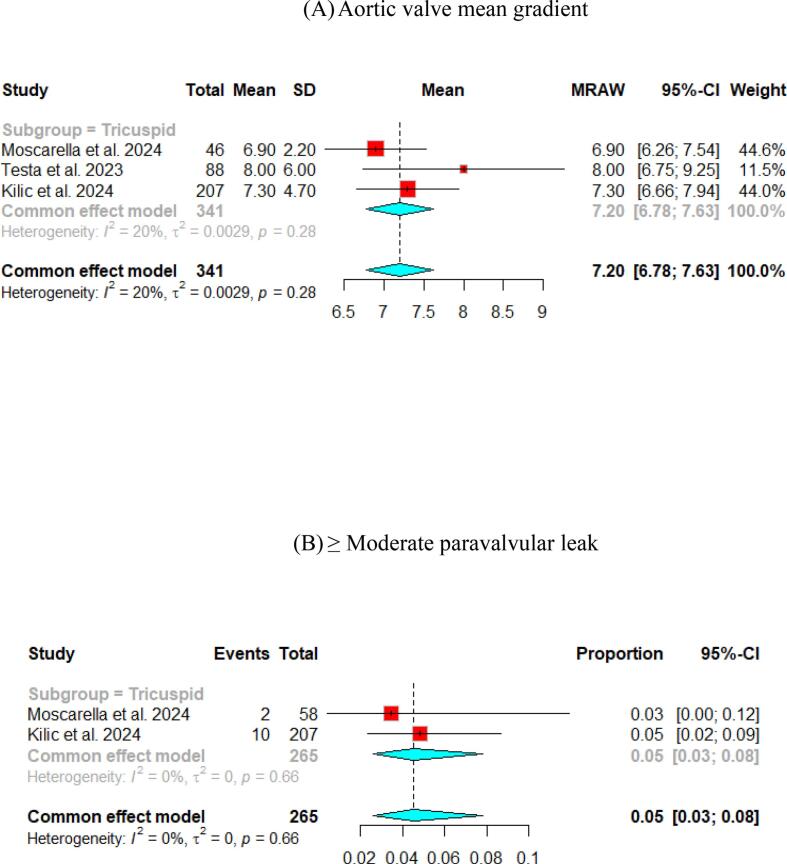
Hemodynamic outcomes of Myval THV in patients with severe aortic stenosis at 2 years:(A) Aortic valve mean gradient, (B) Moderate paravalvular leak.

Fewer studies have experienced long-term follow-up of participants. The Mean aortic valve pressure gradient was 10.63 (95 % CI [9.12, 12.14]) and 7.2 (95 % CI [6.78, 7.63]) at one- and two-year, respectively. Cases with ≥ moderate paravalvular leak were identified in 4 % (95 % CI [2], [7]) at the one-year follow-up and 5 % (95 % CI [3,8]) at the two-year follow-up (Fig. 4, Fig. 5).

### 3.7 The quantitative videodensitometic assessment of aortic regurgitation following TAVI in severe aortic stenosis

A study by Elkoumy et al., 2023[31] investigated the incidence of AR utilizing the validated quantitative Videodensitometry angiography technology. Their study included an analysis of 122 aortograms from 103 patients. Of these patients, 64 (62 %) had TAV, 38 (37 %) had BAV, and one had a unicuspid aortic valve. The incidence of moderate or greater AR was 1.9 %, mild AR was present in 20.4 % of patients, and 77.7 % had no or trace AR. Notably, the two cases with moderate or greater AR were observed in the BAV group**.**

Kawashima et al., 2021[20] performed a comparative study on quantitative angiographic AR after TAVI using three balloon-expandable valves. The research involved 108 patients treated with the Myval valve. In this group, 2.8 % experienced moderate or severe AR, 47.2 % had mild AR, and 50.0 % had no or trace AR. The mean quantitative AR for these patients was 6.3 % ± 6.3 %.

Another study by Abdelshafy et al., 2022[29] which is a comparative study on the incidence of acute AR following TAVI across 11 commercially available TVHs. This study involved data from 108 patients treated with the Myval THV. Findings revealed that 2.8 % of the patients in the Myval group experienced moderate or severe AR, 47.2 % had mild AR, and 50.0 % had no or trace AR. Additionally, PVL was evaluated at discharge or 30-day post-procedure in 103 patients treated with Myval. Results indicated that none of the patients had moderate or severe PVL, 37.9 % had mild PVL, and 62.1 % had no or trace PVL.

**Fig. 6:**
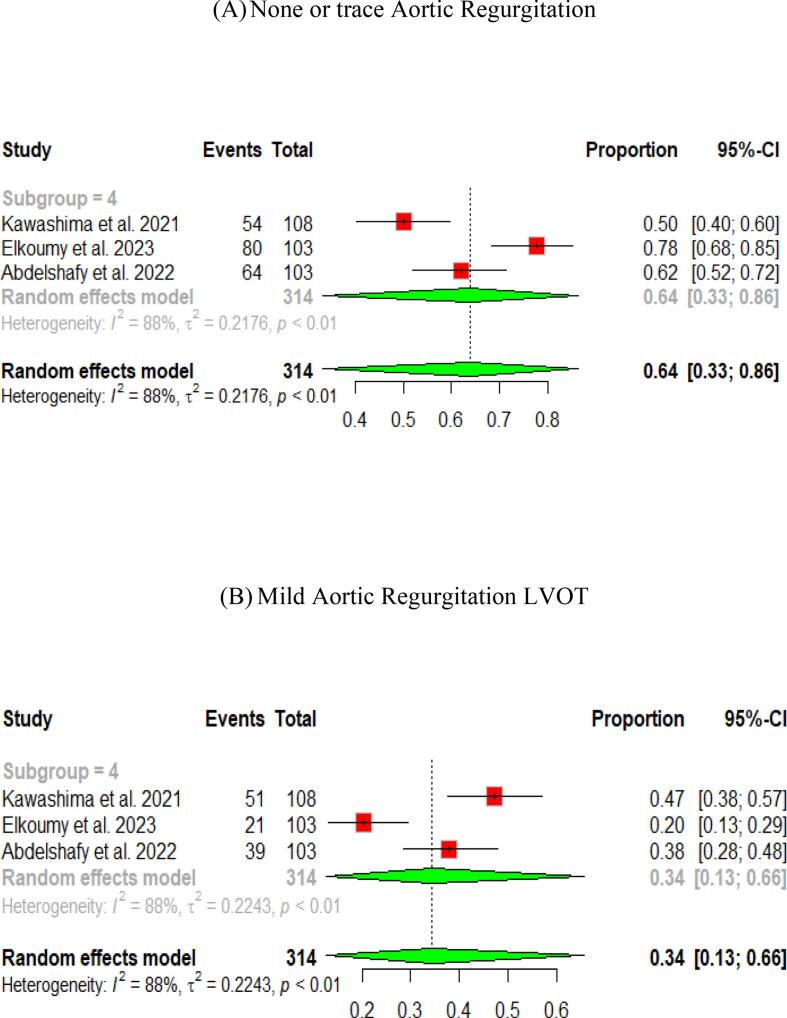

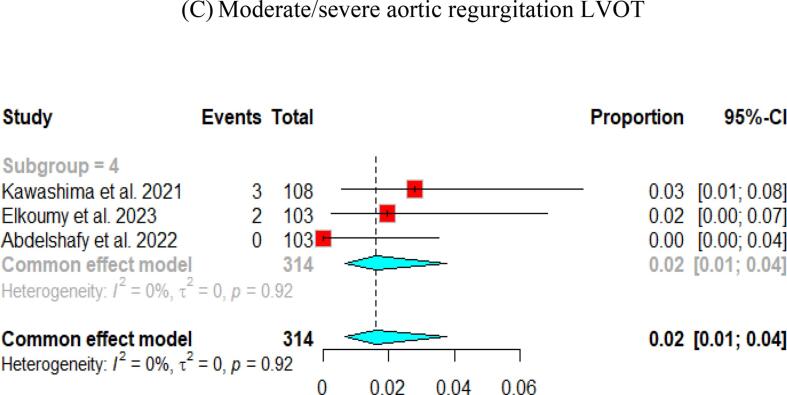
Quantitative angiographic assessment of aortic regurgitation following Myval THV in severe aortic stenosis.

Pooled results of the three studies showed that no/trace AR with quantitative videodensimetry was found in 64 % (95 % CI [33, 86]), while mild AR in 34 % (95 % CI [13, 66]). Patients with moderate/severe AR on videodensimetry were only observed in 2 % (95 % CI [1], [4]) (Fig. 6).

### 3.8 Technical success, device success, early safety, and clinical efficacy of the Myval THV after TAVI in patients with severe aortic stenosis

The pooled technical success and device success reached 97 % (95 % CI [92, 99]) and 97 % (95 % CI [94, 98]), respectively, with the Myval THV (Fig. 2, Fig. 3). Furthermore, we reported the early safety and clinical efficacy, which were 87 % (95 % CI [73, 95]) and 86 % (95 % CI [54, 97]), respectively (Fig. 3, Fig. 4).

### 3.9 Safety and complications outcomes of the Myval THV

The procedural information of Myval THV showed good safety with a pooled procedural death rate of 0 % (95 % CI [0,1]), and the pooled rate of major vascular complication was 4 % (95 % CI [2], [8]) (Table 3 and Supplementary Fig. 1).

**Table 3:** Pooled percentage complications outcomes at discharge, 30-days, 1-year and 2-year of patients who underwent TAVI using the Myval THV.

**Follow up**	**Discharge**	**30 days**	**1-year**	**2-year**
**Complications outcomes**				
All-cause mortality	1 %	3 %	9 %	15 %
Cardiovascular mortality	1 %	2 %	4 %	8 %
Non-cardiovascular mortality	0 %	0 %	5 %	7 %
All stroke	1 %	1 %	5 %	3 %
New permanent pacemaker implantation (PPI)	12 %	11 %	9 %	11 %
Vascular complication (All types)	10 %	10 %	−	−
Major Vascular complication	3 %	3 %	−	−
Minor Vascular complication	6 %	7 %	−	−
Acute kidney injury (stages 2, 3, and 4)	1 %	2 %	5 %	−
Acute kidney injury	2 %	3 %	2 %	3 %
Bleeding (All types)	−	6 %	−	−
Bleeding (type 3 and 4)	2 %	2 %	−	−
Myocardial infarction	0 %	0 %	1 %	0 %
New onset atrial fibrillation	3 %	7 %	−	−
Surgery or intervention related to the device	0 %	1 %	−	−
TAVI-related rehospitalisation	0 %	2 %	−	−
Other cardiovascular rehospitalisation	−	0 %	1 %	−
Non-cardiovascular rehospitalisation	−	−	3 %	−

**Abbreviation:** TAVI; transcatheter aortic valve implantation, THV; transcatheter heart valve.

At discharge, the pooled all-cause mortality, cardiovascular mortality, and stroke were 1 % for each. Meanwhile, the pooled new permanent pacemaker implantation rate was 12 % (95 % CI [6], [23]). Other detailed outcomes and complications are in Table 3 and Supplementary Fig. 2.

The 30-day visit showed comparable results to the discharge visit. It showed that the pooled all-causes mortality was 3 % (95 % CI [2], [4]), and cardiovascular mortality was 2 % (95 % CI [1], [3]). The incidence of stroke was low (1 %), and the pooled percentage of patients who had permanent pacemaker implantation was 11 % (95 % CI [7], [15]). (Table 3 and Supplementary Fig. 3).

At the one-year follow-up, the pooled all-cause mortality increased up to 9 % (95 % CI [7], [11]), and cardiovascular mortality increased to 4 % (95 % CI [3], [6]). Also, the pooled percentage of patients with stroke was 5 % (95 % CI [3], [7]). However, at the 2-year follow-up, the pooled all-cause mortality increased to 15 % (95 % CI [12], [19]) and cardiovascular mortality to 8 % (95 % CI [6], [12]). Further details are available in Table 3, Supplementary Figs. 4 and 5.

### 3.10 Transcatheter valve-in-valve (ViV) and non-calcified aortic regurgitation (NCAR) with the Myval THV

The study by Moscarella et al., 2023[34] evaluated the use of Myval THV in a Transcatheter (ViV) procedure in 33 patients with severe symptomatic aortic BHV failure. The results showed that Myval THV reduced the mean transvalvular gradient to 11.6 ± 4.4 mmHg, and a 15-month follow-up revealed a 97 % survival rate among them.

Another study by Ielasi et al., 2021 documented the outcomes of using the BE-MyVal THV in ViV procedures for five patients experiencing BHV failure. No complications were observed in either of these patients. Two patients required blood transfusions due to procedural bleeding, and one patient experienced a minor post-implantation paravalvular leak and needed surgical intervention for an apical left ventricle pseudoaneurysm. The patients' in-hospital stays ranged from 6 days to 2 weeks, with no device failures or major complications reported during this time.

Sanchez-Luna et al., 2023 assessed the safety and feasibility of Myval in 113 patients with NCAR. Aortic root dilatation was observed in 59.3 % of patients, 7.1 % had bicuspid valves, and the mean annular area was 638.6 ± 106.0 mm2. The procedure's technical success rate was 94.7 %, with an 8.9 % rate of residual ≥ moderate AR and a 22.2 % pacemaker implantation rate. There were no incidents of annular rupture, cardiac tamponade, or aortic dissection, although valve embolization occurred in 3.5 % of patients (4 cases), all with a tapered left ventricle outflow tract (p = 0.007). Mortality rates at 30-day and 1 year were 5.3 % and 9.7 %, respectively. Technical success correlated with improved survival (97.1 % vs. 72.7 %; p = 0.012), and valve embolization was a significant determinant of mortality (p = 0.047).

The BE-PANTHEON study by Poletti et al., 2024 compared the Myval THV vs Sapien THV in patients with pure native aortic valve regurgitation (NAVR). The results were comparable with Sapien THV. Myval THV had a device success of 90 % compared to 81 % in Sapien THV (p > 0.1).

## 4 Discussion

This *meta*-analysis of Myval THV summarized all studies that used Myval in patients with severe aortic stenosis and the off-label usage in transcatheter ViV and pure NAVR. Also, it provides detailed information on hemodynamics per aortic valve morphology and study population.

The main findings of this *meta*-analysis were: (1) good hemodynamic upon discharge, 30-day, and longer follow-up. (2) The Myval THV is safe because of the low procedural events following TAVI and the low complication rate during follow-up. (3) higher percentage of technical success, device success, and clinical efficacy across in patients with severe aortic stenosis (4) Lower incidence of ≥ moderate AR with echocardiography and videodensitometry. 5) Myval THV is an alternative option for off-label use during transcatheter (ViV) and patients with pure NAVR.

The Myval THV is a novel expandable valve that is considered an alternative option to the Sapien THV. Its unique design and intermediate sizes provide a good option during TAVI for patients with severe aortic stenosis[11], [33]. Furthermore, the off-label use of Myval THV was observed in patients with stenotic bicuspid aortic valve[22], [23], [36], [41], large annulus[38], transcatheter ViV[21], [34], and in pure NAVR[32], [45].

Additionally, quantitative videodensitometry is a novel technique used for assessing AR. This meta-analysis reported a pooled incidence of mild AR using quantitative videodensitometry in 34 %, while moderate/severe AR was estimated at 2 %. The LANDMARK trial reported a 3 % incidence of moderate/severe AR in the Myval THV, giving closer results to the quantitative videodensitometry in this *meta*-analysis.

Another essential aspect following TAVI is the hemodynamic and safety outcomes. Following TAVI, the pooled aortic valve mean gradient was 9.25 mmHg and reported as 8.46 mmHg at 30 days. The pooled results of the long-term follow-up were 10.63 mmHg at one year and 7.2 mmHg at two years. In the LANDMARK trial[14], the results confirmed the non-inferiority of Myval THV against Sapien and Evolut THV and found that the mean gradient of the aortic valve was 8.31 mmHg. Other studies included in the forest plot at 30-day of mean aortic pressure gradient showed a range from 6 – 10.4 mmHg (Fig. 3b). This fact confirms the efficacy of the Myval THV in reducing the mean aortic pressure gradient following TAVI. In the low-risk trial using Evolut THV in patients with severe aortic stenosis compared to the surgical aortic-valve replacement[47], a long-term follow-up was conducted for up to two years, revealing a mean aortic valve pressure gradient of 10.5, 11.2, and 12.3 mmHg at 30 days, one year, and two years, respectively, in the Evolut THV group. These findings were similar in this *meta*-analysis of the pooled mean aortic valve pressure gradient of Myval THV at long-term follow-up.

Moreover, this *meta*-analysis reported a low percentage of moderate AR following discharge, 30-day, one year, and two years. Additionally, the Myval THV improved the aortic valve area after TAVI reached 1.93 and 1.95 cm^2^ at discharge and 30-day, respectively. The results were consistent with the LANDMARK, which reported an aortic valve area of 2.16 and 2.02 cm^2^ at discharge and 30 days, respectively.

The safety of the TAVI plays an important role and should be considered during the procedure. The Myval THV demonstrated high technical success, device success, and clinical efficacy across the included studies.

In addition to TAVI, the off-label usage of Myval THV was seen in pure NAVR[32], [45], which showed safety and was deemed an alternative. In addition, it has been used in large aortic annulus[38], which showed that only 3 out of 10 had mild AR and had good outcomes after TAVI. Extending the importance of Myval THV to the transcatheter ViV[21], [34], showing it as a good option and showed a high success rate besides the good outcomes.

This first *meta*-analysis provides detailed information about the Myval balloon-expandable valve. It included many studies using Myval THV for treating patients with severe aortic stenosis and off-label usage in large annulus, bicuspid valve, transcatheter ViV, and AR. It also included studies that assessed AR. Furthermore, it provided information on hemodynamic outcomes, safety, and complications following TAVI, 30-day, one-year, and two-year follow-ups.

One of the limitations of this study is the lack of long-term follow-up randomized clinical trials to show the efficacy of Myval THV. However, this will be resolved and will be reported in the one-year follow-up of the LANDMARK trial[14]. Also, a limited number of studies reported the off-label usage of Myval THV for patients with pure NAVR and during transcatheter ViV and usage during TAVI in special populations of severe aortic stenosis. The large number of included studies from different countries contributed to increased heterogeneity.

## 5 Conclusion

The Myval THV is considered a good option for patients undergoing TAVI, and has demonstrated favorable hemodynamic performance and safety in short and long-term patients with aortic stenosis. However, additional long-term follow-up trials are mandatory to assess its real impact on patients with severe aortic stenosis.

**Funding**.

This study received no funding.

## CRediT authorship contribution statement

**Elfatih A. Hasabo:** Writing – review & editing, Writing – original draft, Visualization, Validation, Software, Resources, Project administration, Methodology, Investigation, Data curation, Conceptualization. **Amira A. Aboali:** Writing – review & editing, Writing – original draft, Validation, Resources, Project administration, Methodology. **Lina Hemmeda:** Writing – review & editing, Writing – original draft, Methodology, Data curation. **Ammar Elgadi:** Writing – review & editing, Writing – original draft, Formal analysis. **Salma S. Alrawa:** Writing – review & editing, Writing – original draft, Data curation. **Alaa S. Ahmed:** Writing – review & editing, Data curation. **Malaz M. Abdalmotalib:** Writing – review & editing, Visualization, Data curation. **Abdullatif Yasir H Eissa:** Writing – review & editing, Visualization, Data curation. **Mohammed Mahmmoud Fadelallah Eljack:** Writing – review & editing, Writing – original draft, Visualization. **Sherif Sultan:** Writing – review & editing, Supervision. **Osama Soliman:** Writing – review & editing, Supervision.

## Declaration of competing interest

The authors declare that they have no known competing financial interests or personal relationships that could have appeared to influence the work reported in this paper.
