# Supplementary material for: A systematic review and meta-analysis of the hemodynamics and outcomes of the Myval balloon-expandable valve in patients with severe aortic stenosis and with aortic regurgitation
Source: Int J Cardiol Heart Vasc. 2025 Mar 6;58:101641. doi: 10.1016/j.ijcha.2025.101641 (PMC11930208; doi:10.1016/j.ijcha.2025.101641)

# **Supplementary Table 1:** Search strategy for included studies

| PubMed | ("Myval"[All Fields] AND "Octacor"[All Fields]) OR "Myval"[All Fields] | 69 |
| --- | --- | --- |
| Scopus | ( TITLE-ABS-KEY ( Myval ) OR TITLE-ABS-KEY ( Myval AND octacor ) ) | 119 |
| Web Of Science | Myval Octacor (All Fields) or Myval (All Fields) | 75 |
| Cochrane | (Myval OR “Myval Octacor”) | 8 |
| Embase | (Myval OR “Myval Octacor”) | 175 |
| **Total** |  | 446 |

# **Supplementary Table 2:** Risk of Bias assessment for the included studies using ROBBINS-I tool

| Study | Bias due to confounding | Bias in selection of participants into the study | Bias in classification of interventions | Bias due to deviations from intended interventions | Bias due to missing data | Bias in measurement of outcomes | Bias in selection of the reported result | Overall Risk of Bias |
| --- | --- | --- | --- | --- | --- | --- | --- | --- |
| Sharma et al. 2020 (1) | Moderate | moderate | low | low | Moderate | low | Moderate | Moderate |
| Kawashima et al. 2021 (2) | Moderate | Critical | Critical | Moderate | Critical | low | moderate | critical |
| Ielasi et al. 2021 (3) | critical | critical | No information available | No information available | critical | moderate | moderate | critical |
| Elkoumy et al. 2022 and 2023 (4, 5) | Moderate | Moderate | Low | Low | Low | Low | Low | Moderate |
| García-Gómez et al. 2022 (6) | Moderate | Moderate | Low | Low | Low | Low | Low | Moderate |
| Halim J et al. 2022 (7) | Moderate | Low | Low | Low | Low | Low | Low | Moderate |
| Delgado-Arana JR, et al. 2022 (8) | Low | Low | Low | Low | Moderate | Low | Low | Low |
| S. Santos-Martinez et al. 2022 (9) | Serious | Moderate | Low | Moderate | Moderate | Moderate | Moderate | Serious |
| Akyüz et al. 2022 (10) | Moderate | Moderate | Low | Moderate | Moderate | Moderate | Moderate | Moderate |
| Abdelshafy et al. 2022 (11) | Moderate | Serious | Moderate | Low | Low | Low | No Information Available | Serious |
| Barki et al. 2022 (12) | Moderate | Low | Low | Moderate | Moderate | Moderate | Low | Moderate |
| Elkoumy et al. 2023 (13) | Low | Low | Low | Low | Moderate | Moderate | Low | Moderate |
| Sanchez-Luna et al. 2023 (14) | Moderate | Low | Low | Moderate | Moderate | Low | Moderate | Moderate |
| Testa et al. 2023 (15) | Moderate | Low | Low | Low | Moderate | Low | Low | Moderate |
| Moscarella et al. 2023 (16) | Moderate | Low | Low | Low | Low | Low | Moderate | Moderate |
| Magyari et al. 2023 (17) | Moderate | Low | Low | Low | Low | Low | Low | Low |
| Amat-Santos et al. 2023 (18) | Moderate | Low | Low | Low | Low | Low | Moderate | Moderate |
| Halim, J et al. 2023 (19) | Moderate | Low | Low | Low | Moderate | Low | Moderate | Moderate |
| Holzamer et al. 2023 (20) | Moderate | Moderate | Low | Moderate | Moderate | Low | Moderate | Moderate |
| Boljevic et al. 2023 (21) | Moderate | Low | Low | Low | Low | Low | Low | Low |
| Moscarella et al. 2024 (22) | Moderate | Low | Low | Moderate | Low | Low | Low | Low |
| Magyari et al. 2024 (23) | Moderate | Low | Low | Low | Moderate | Moderate | Low | Moderate |
| Jose et al. 2024 (24) | Low | Moderate | Low | Low | Moderate | Moderate | Low | Moderate |
| Kilic et al. 2024 (25) | Moderate | Moderate | Low | Low | Moderate | Moderate | Low | Moderate |
| Baumbach et al. 2024 (Landmark) (26) | Low | Low | Low | Low | Moderate | Low | Low | Low |
| Ubben et al. 2024 (27) | Low | Moderate | Low | Low | Moderate | Low | Low | Moderate |
| Poletti et al. 2024 (28) | Low | Moderate | Low | Low | Moderate | Moderate | Low | Moderate |
| Amber et al. 2024 (29) | Moderate | Moderate | Low | Low | Moderate | Moderate | Low | Moderate |

**Supplementary Figure 1:**Procedural outcomes following Myval THV in severe aortic stenosis**:** (A) Procedural Death, (B) Failed delivery, (C) Major Vascular complications, (D) More than one procedure required, (E) Conversion to surgery, (F) Device migration or embolization, (G) Annulus Rupture, (H) Cardiac tamponade, (I) Coronary obstruction, (J) Successful position and implantation

1. **Procedural Death**

**
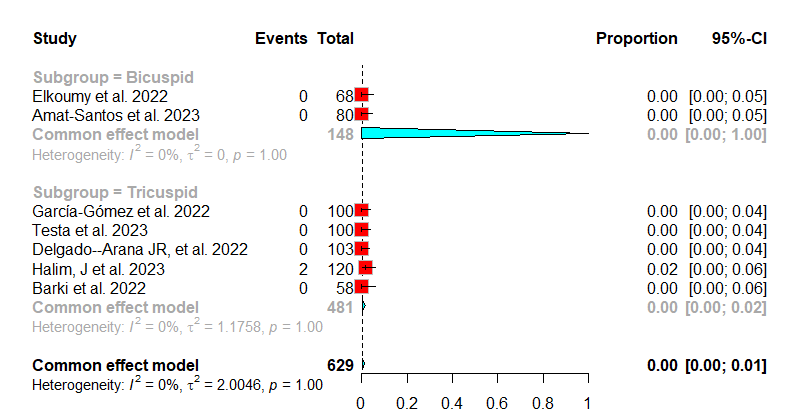
**

1. **Failed delivery**

**
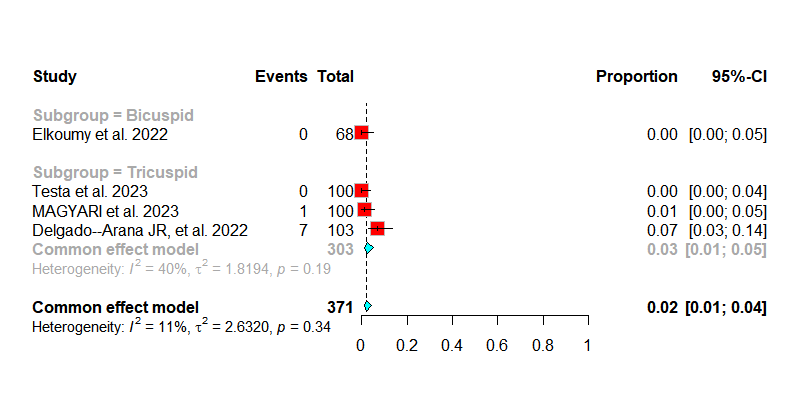
**

1. **Major Vascular complications**

**
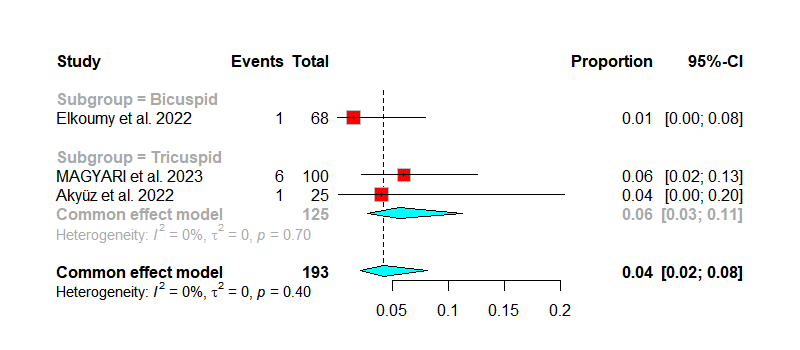
**

1. **More than one prosthesis required**

**
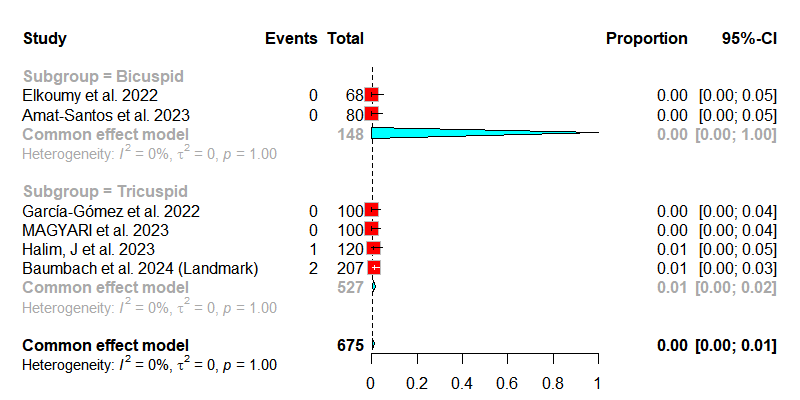
**

1. **Conversion to surgery**

**
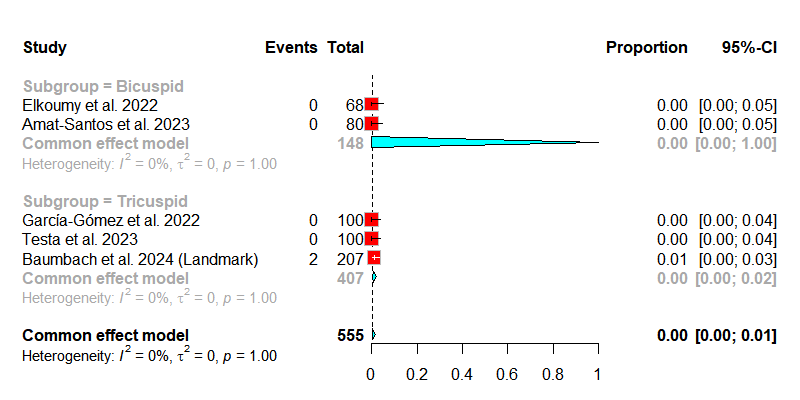
**

1. **Device migration or embolization**

**
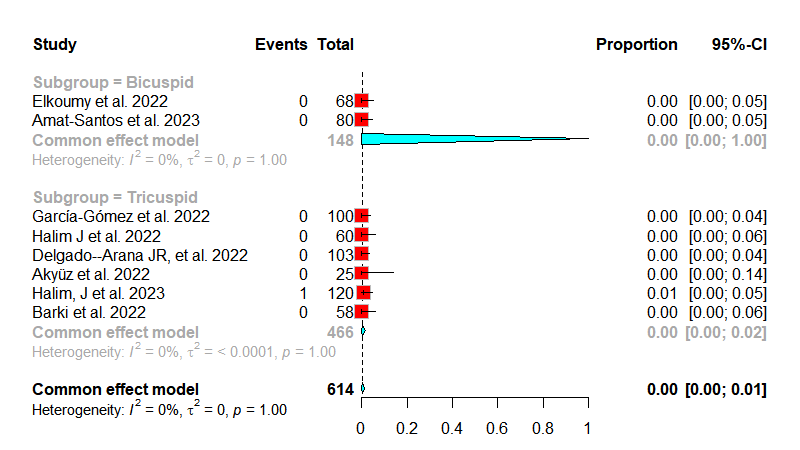
**

1. **Annulus Rupture**

**
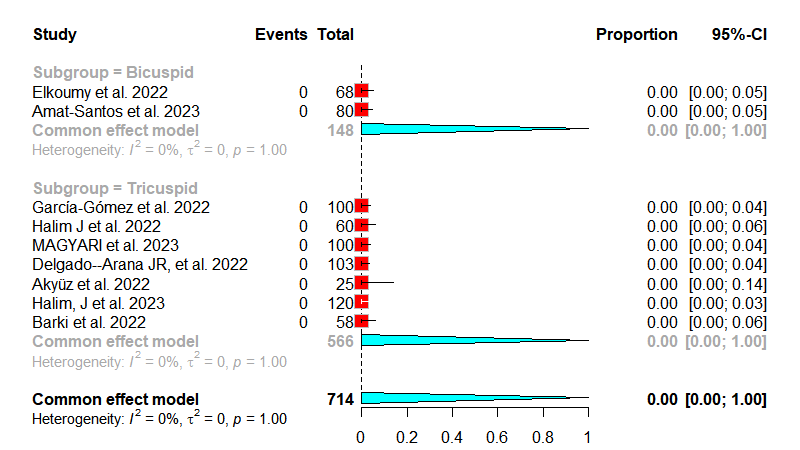
**

1. **Cardiac tamponade**

**
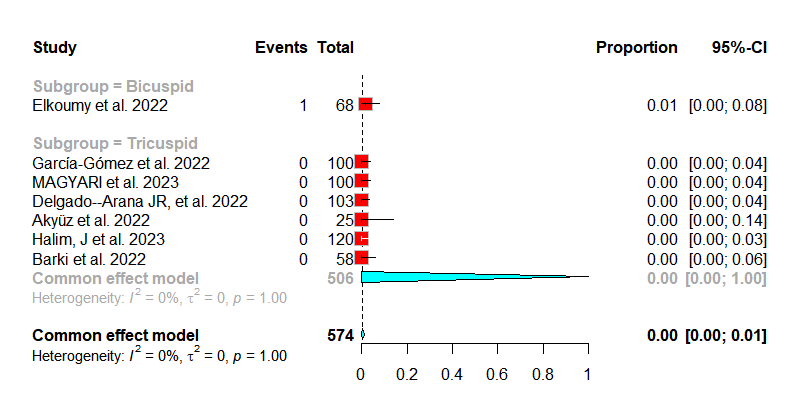
**

1. **Coronary obstruction**

**
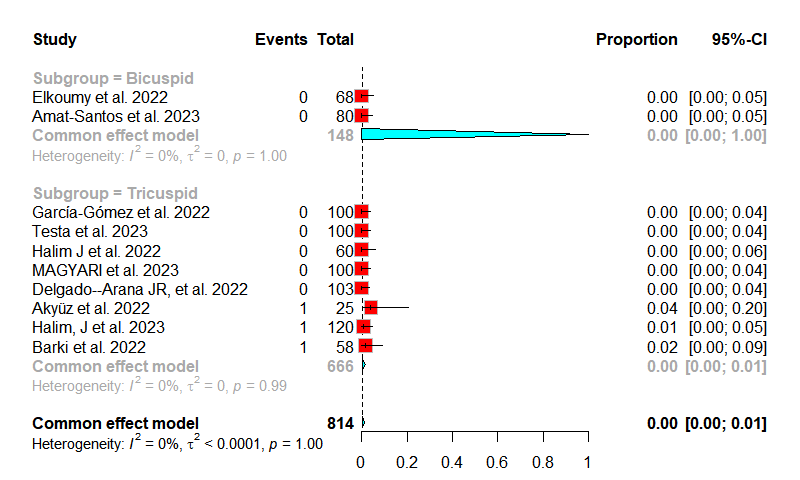
**

1. **Successful position and implantation**

**
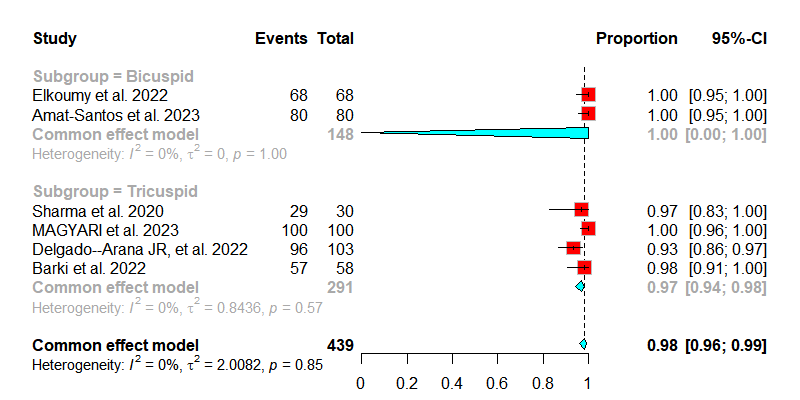
**

**Supplementary Figure 2:** Outcomes following Myval THV in patients with aortic stenosis and aortic diseases **at discharge:** (A) All cases mortality, (B) Cardiovascular mortality, (C) Non cardiovascular mortality, (D) All stroke, (E) New permanent pacemaker implantation, (F) Vascular complications all types, (G) Major vascular complications, (H) Minor vascular complications, (I )Acute kidney injury stages 2,3 and 4, (J) Acute kidney injury, (K) Bleeding type 3 and 4 ,(L) Myocardial infarction, (M) New onset atrial fibrillation, (N) TAVI related rehospitalization

1. **All causes mortality**

**
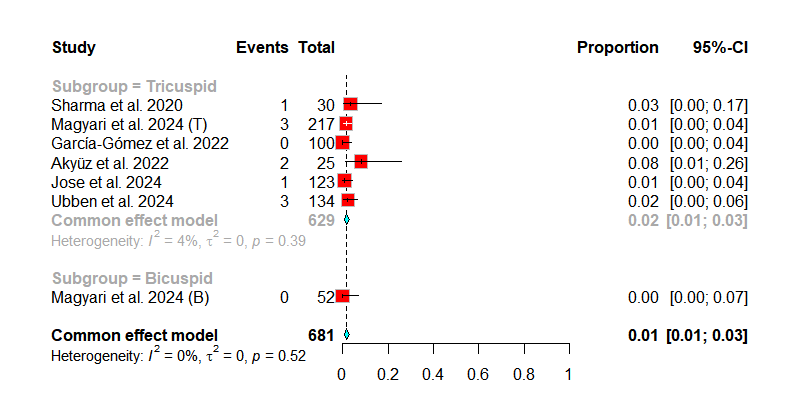
**

1. **Cardiovascular mortality**

**
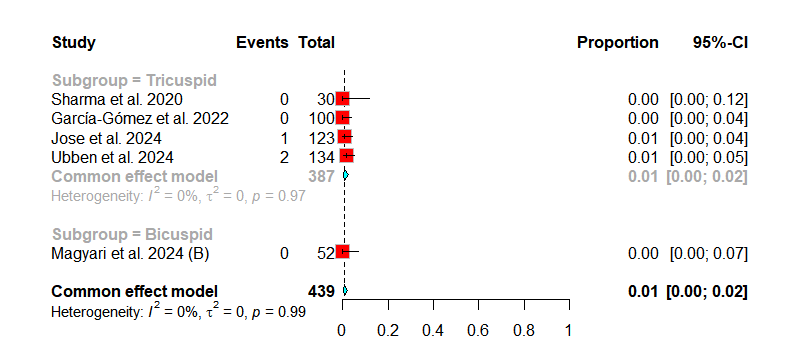
**

1. **Non cardiovascular mortality**

**
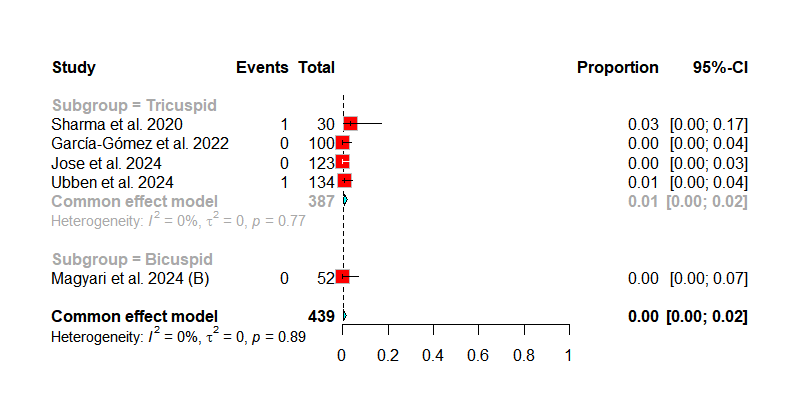
**

1. **All stroke**

**
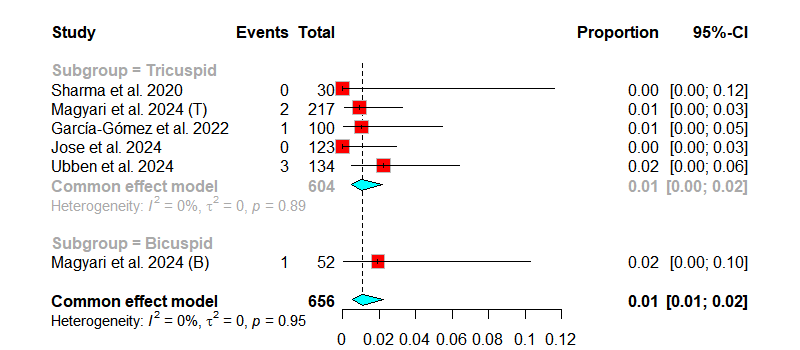
**

1. **New permanent pacemaker implantation**

**
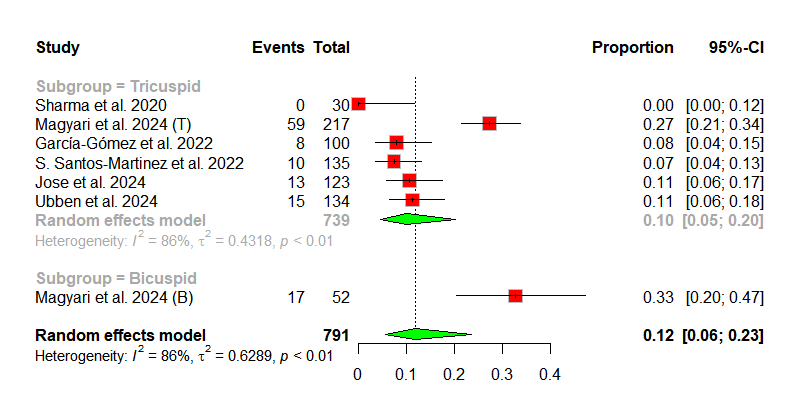
**

1. **Vascular complications all types**

**
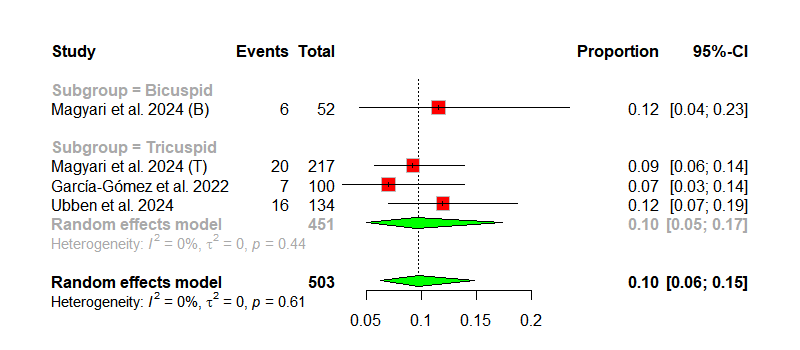
**

1. **Major vascular complications**

**
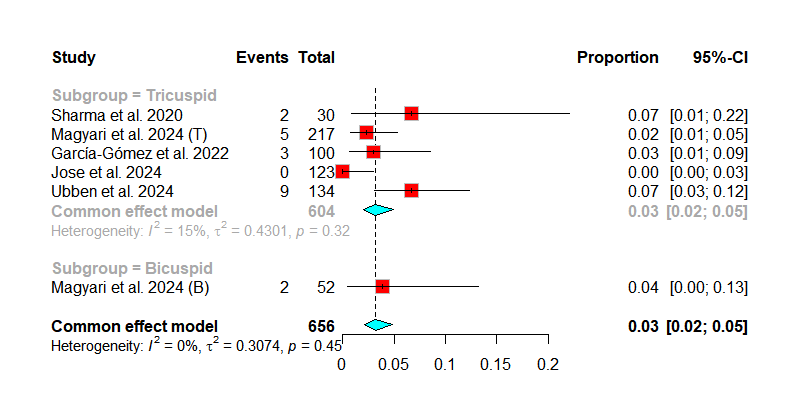
**

1. **Minor vascular complications**

**
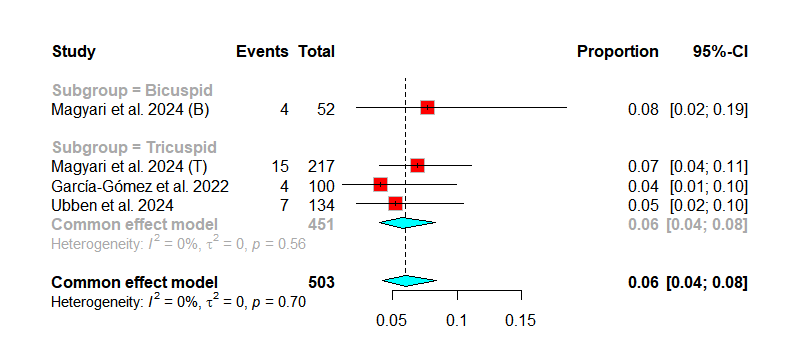
**

1. **Acute kidney injury stages 2,3 and 4**

**
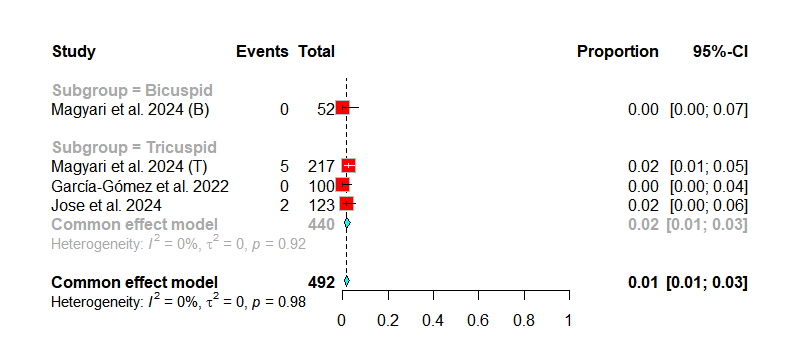
**

1. **Acute kidney injury**

**
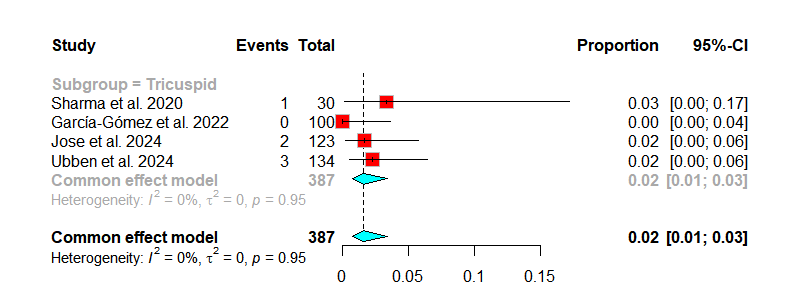
**

1. **Bleeding type 3,4**

**
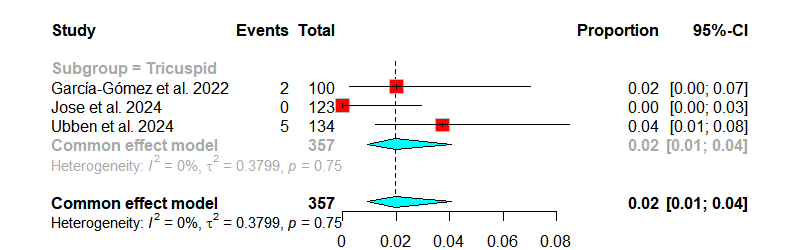
**

1. **Myocardial infarction**

**
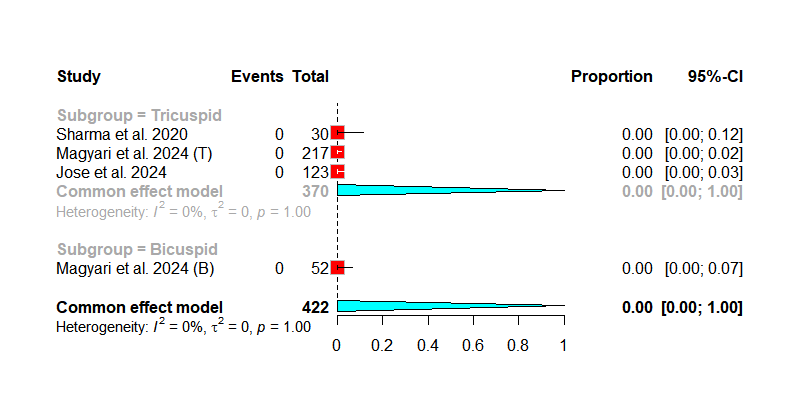
**

1. **New onset atrial fibrillation**

**
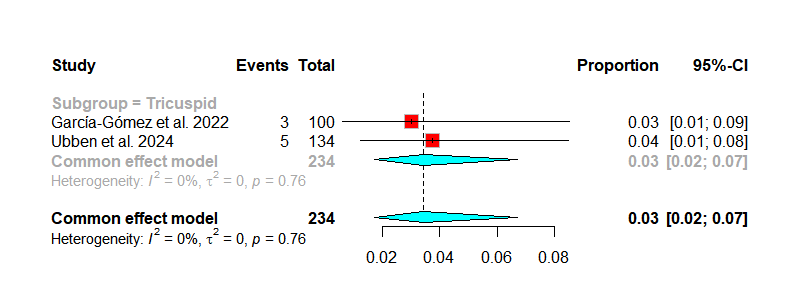
**

1. **TAVI related rehospitalization**

**
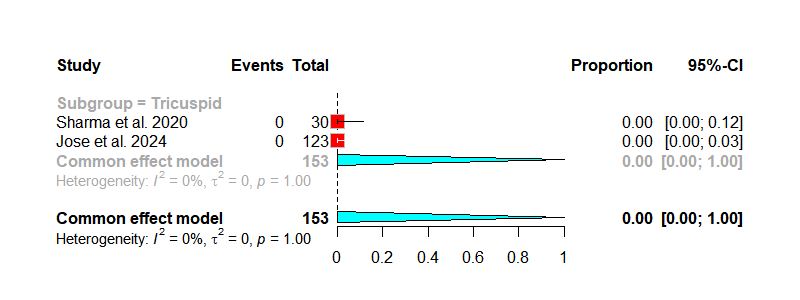
**

**Supplementary Figure 3:** Outcomes following Myval THV in patients with aortic stenosis and aortic diseases **30-day:** (A) All cases mortality, (B) Cardiovascular mortality, (C) Non cardiovascular mortality, (D) All stroke, (E) New permanent pacemaker implantation, (F)Vascular complications all types, (G) Major vascular complications (H) Minor vascular complications, (I)Acute kidney injury stages 2,3 and 4, (J)Acute kidney injury, (K)Bleeding all types, (L) Bleeding type 3,and 4, (M) Myocardial infarction, (N) New onset atrial fibrillation, (O) Surgery or intervention related to device, (P)TAVI related rehospitalization, (Q) Other cardiovascular rehospitalization, (R) Incidence of patients with mean aortic gradient over 20 mmHg

1. **All-causes mortality**


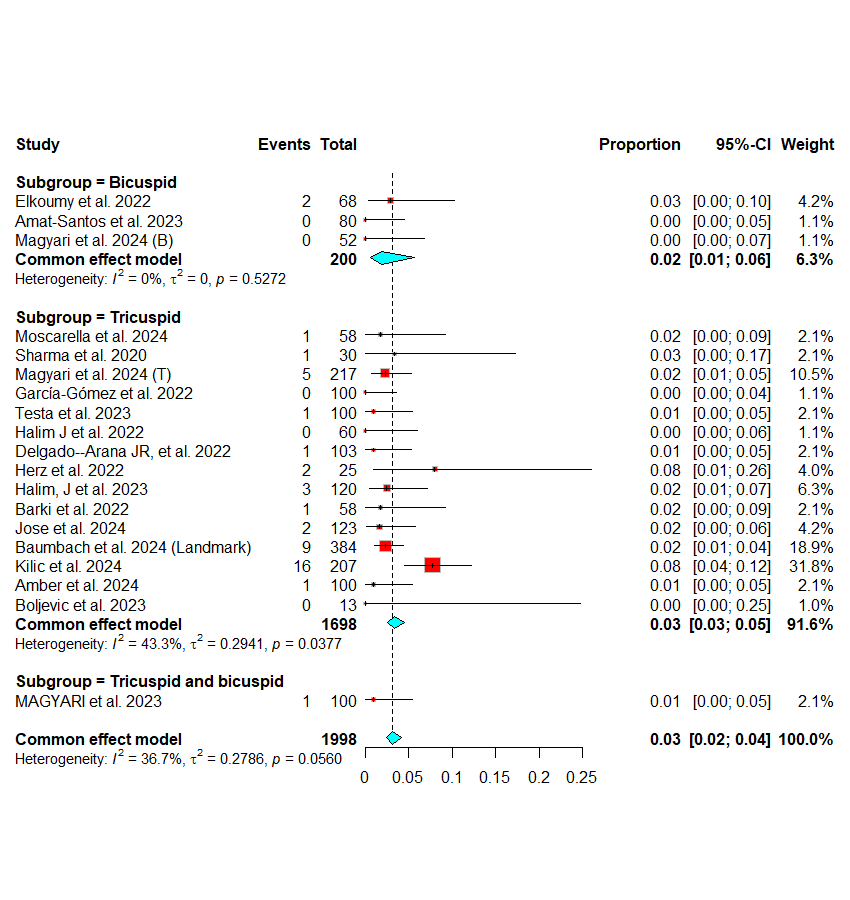


1. **Cardiovascular mortality**


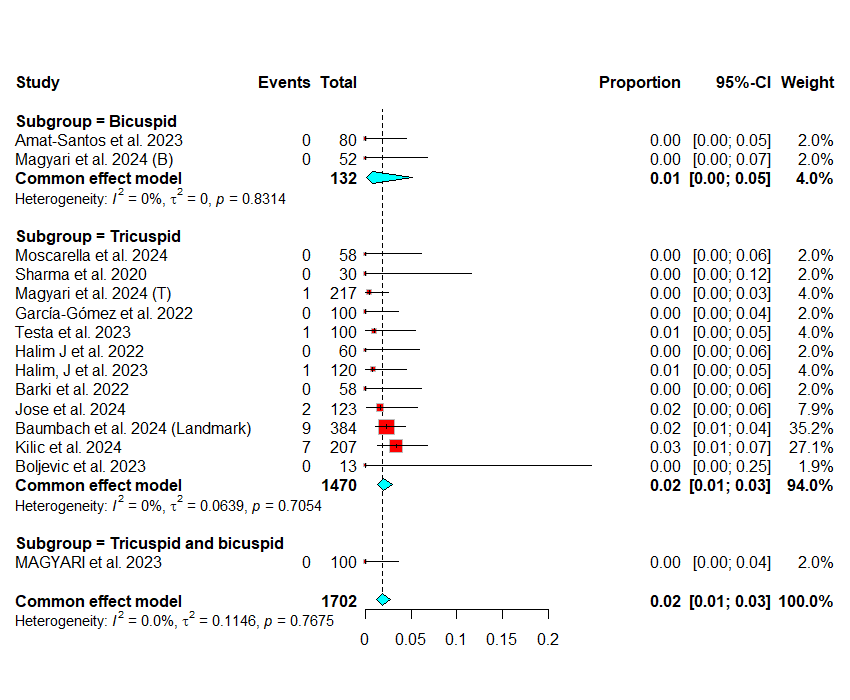


1. **Non cardiovascular mortality**


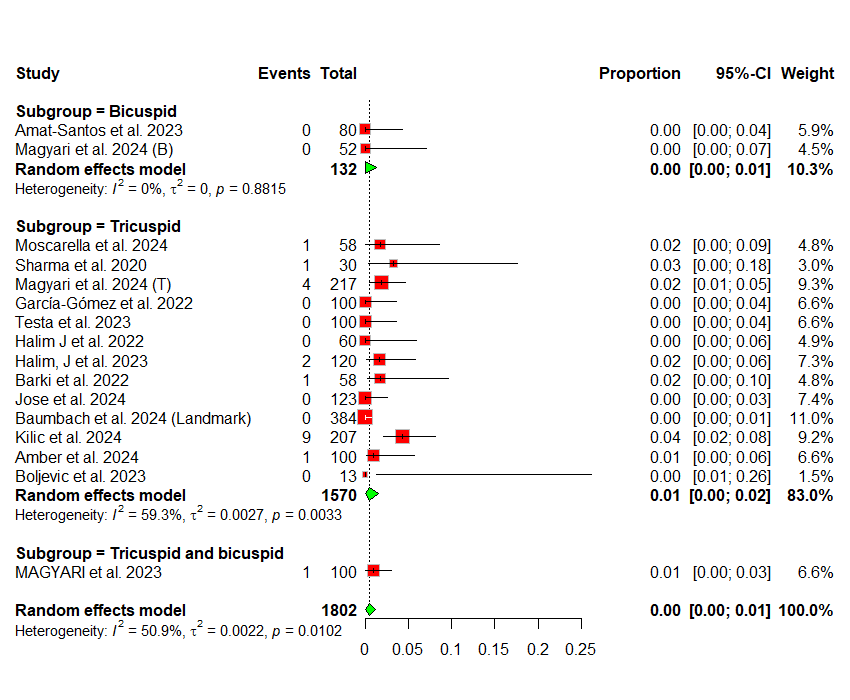


1. **All stroke**


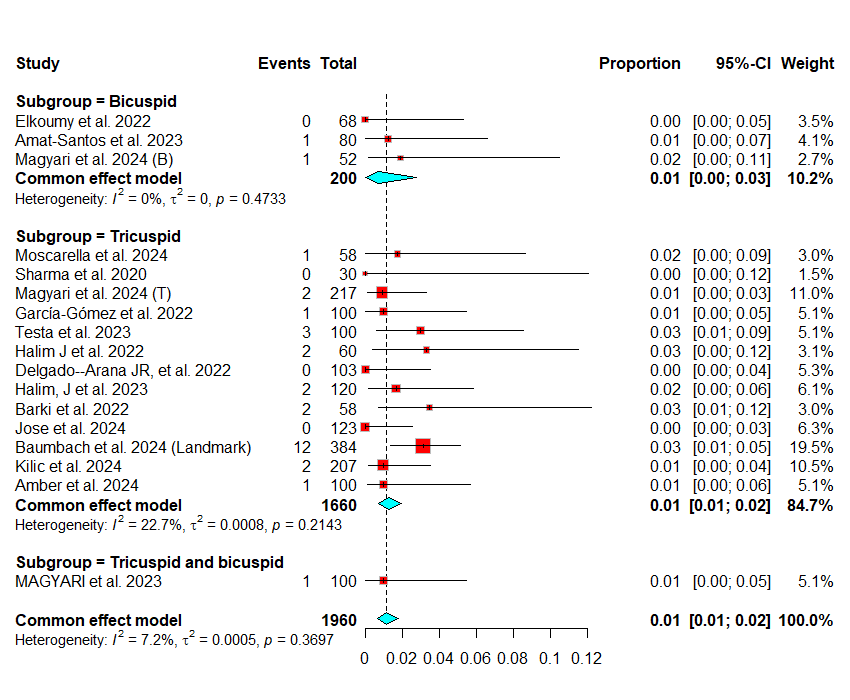


1. **New** **permanent pacemaker implantation**


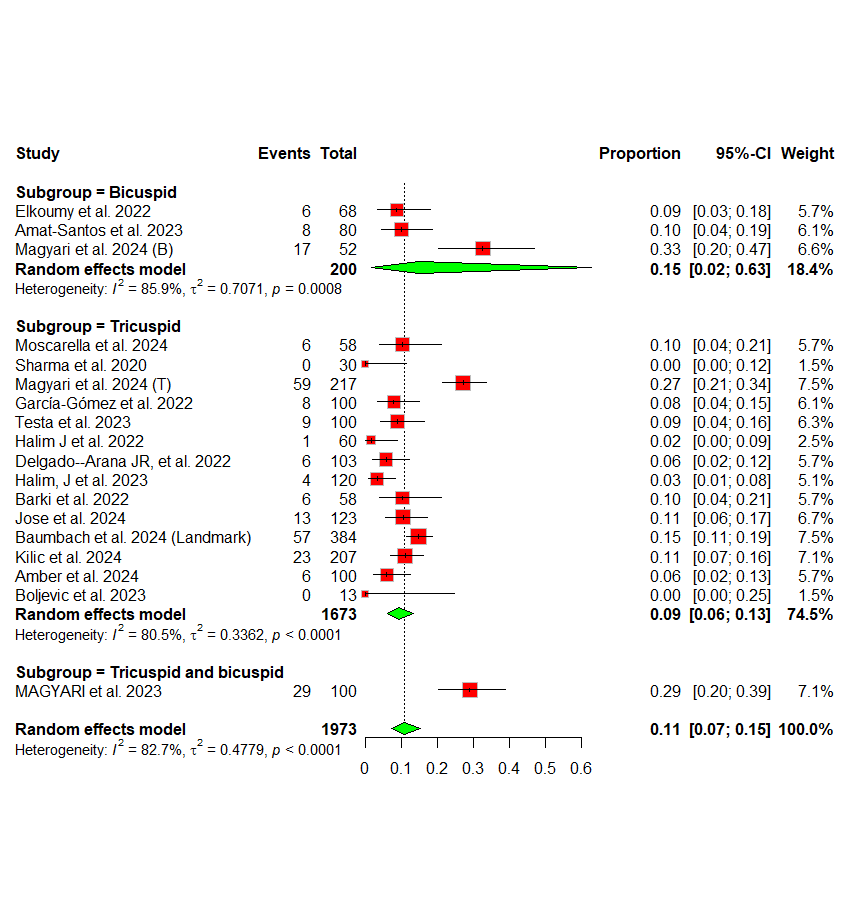


1. **Vascular complications all types**

**
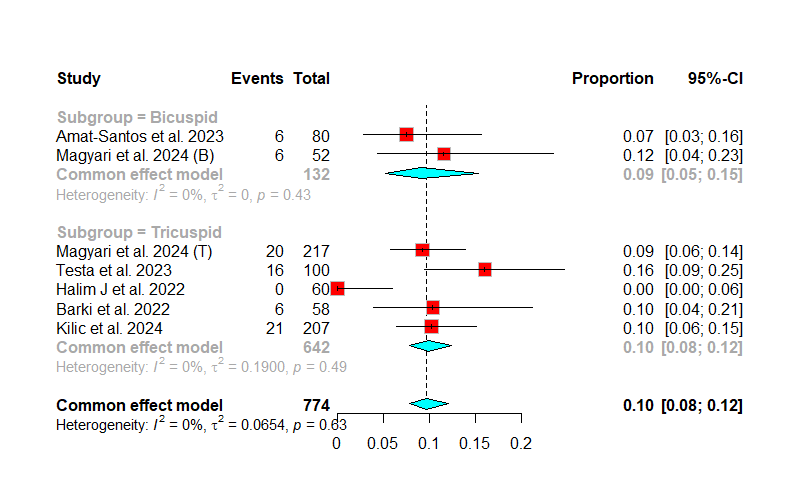
**

1. **Major vascular complications**


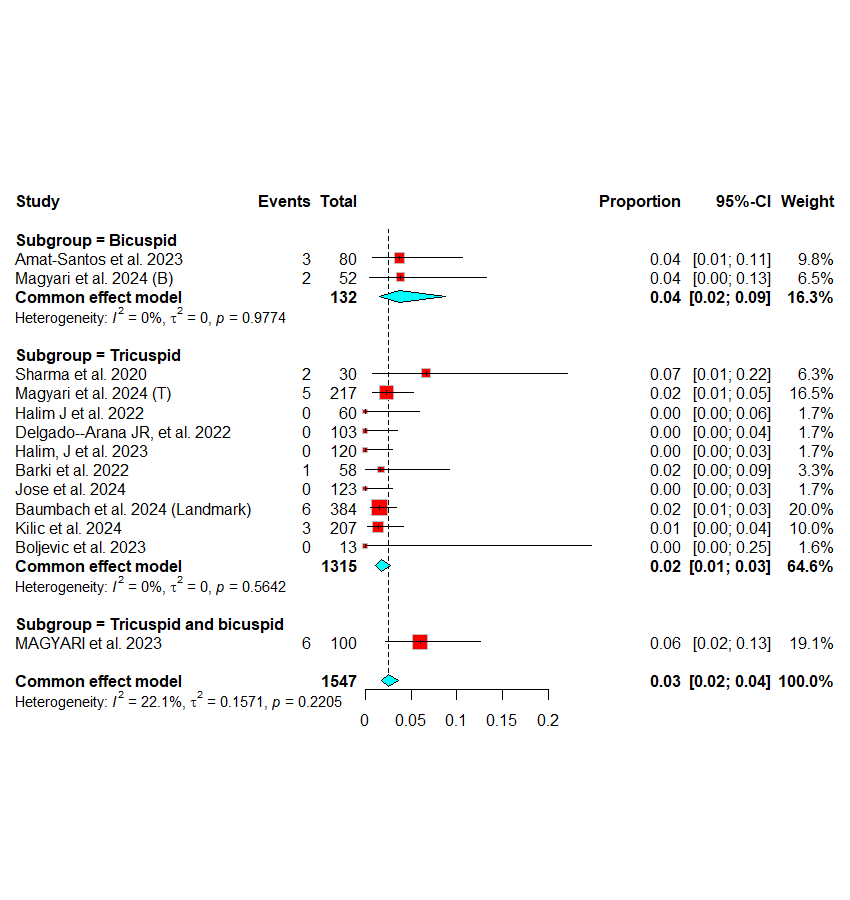


1. **Minor vascular complications**


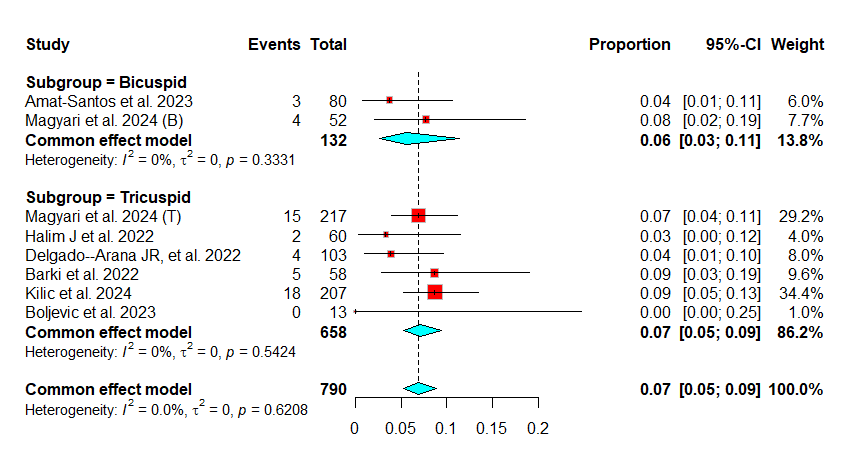


1. **Acute kidney injury stages 2,3 and 4**

**
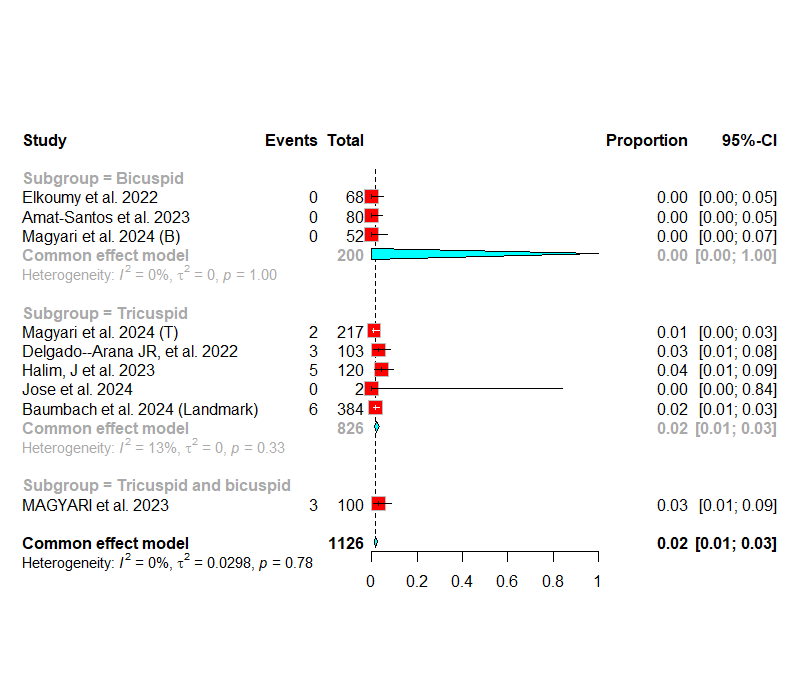
**

1. **Acute kidney injury**


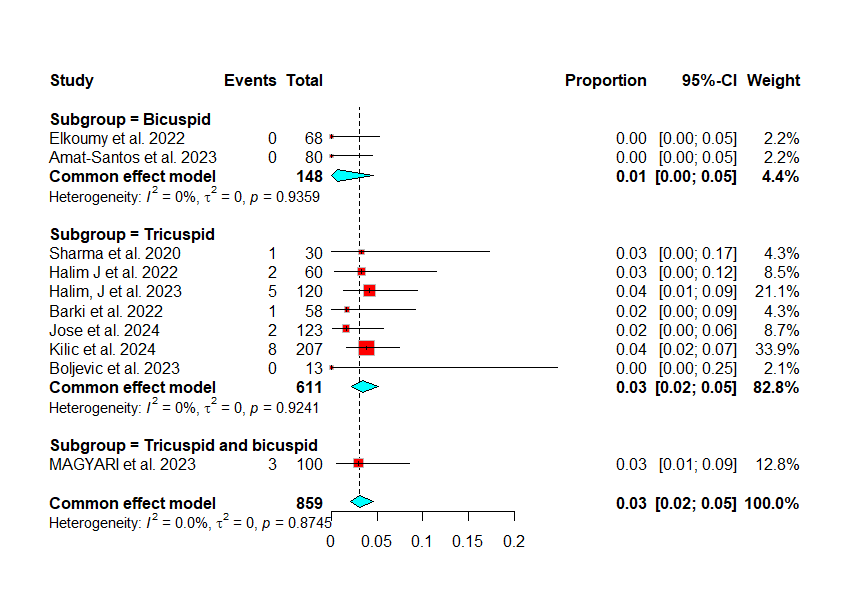


1. **Bleeding all types**


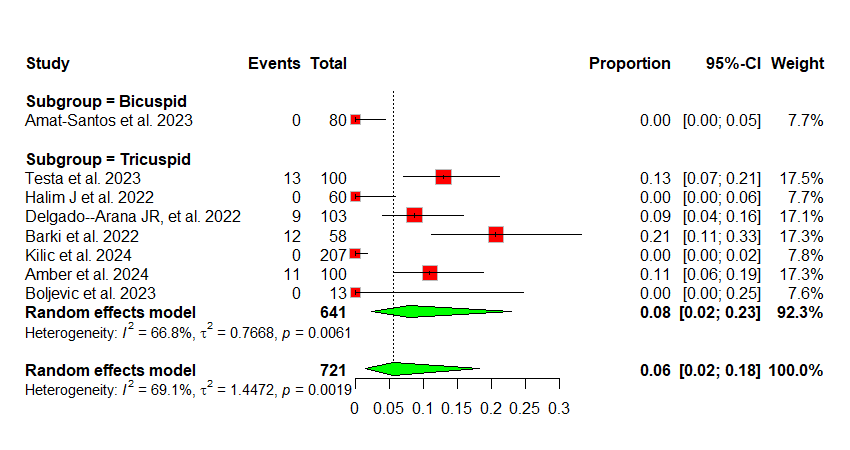


1. **Bleeding type 3 and 4**

**
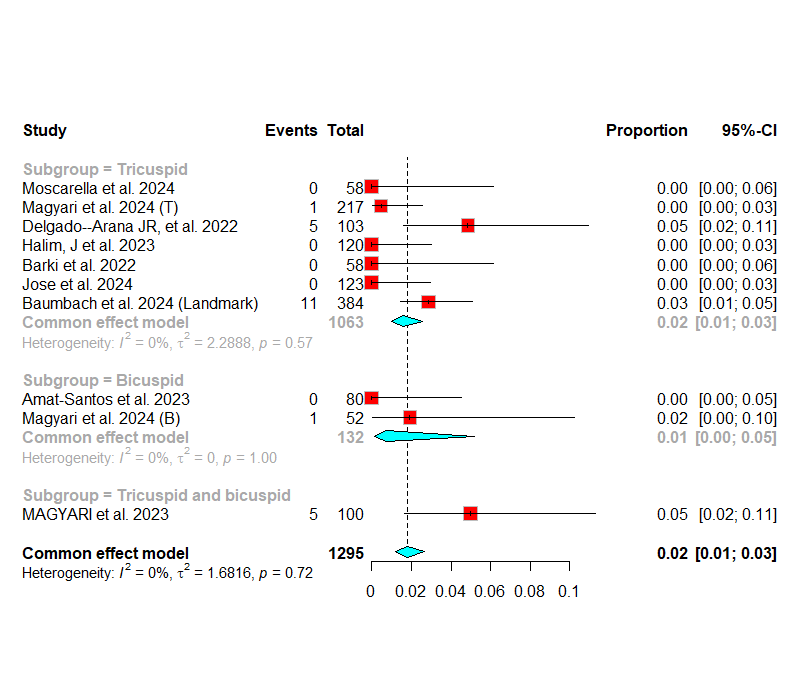
**

1. **Myocardial infarction**

**
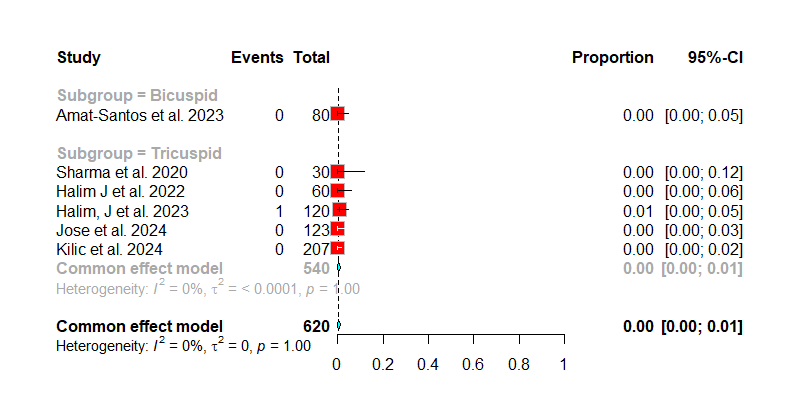
**

1. **New onset atrial fibrillation**


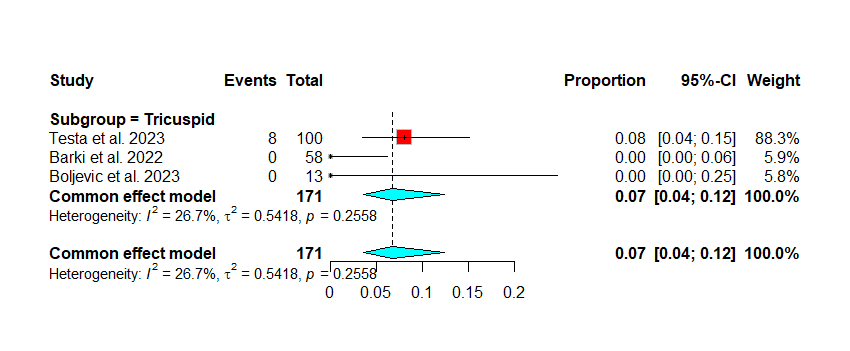


1. **Surgery or intervention related to device**


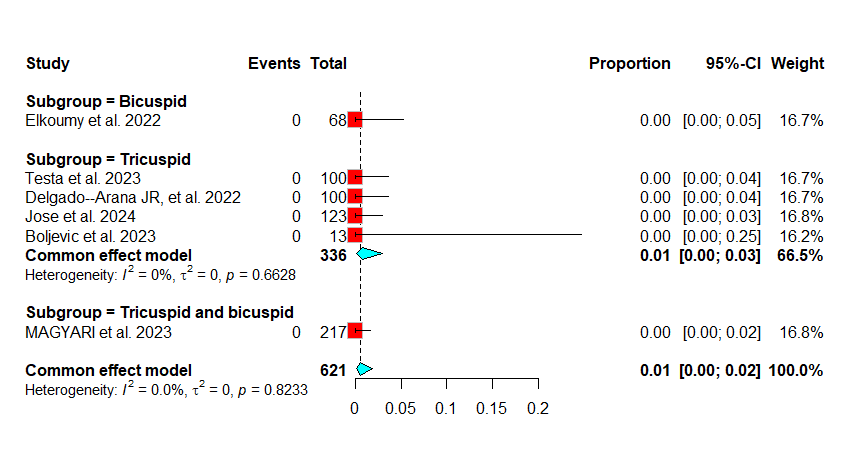


1. **TAVI related rehospitalisation**

**
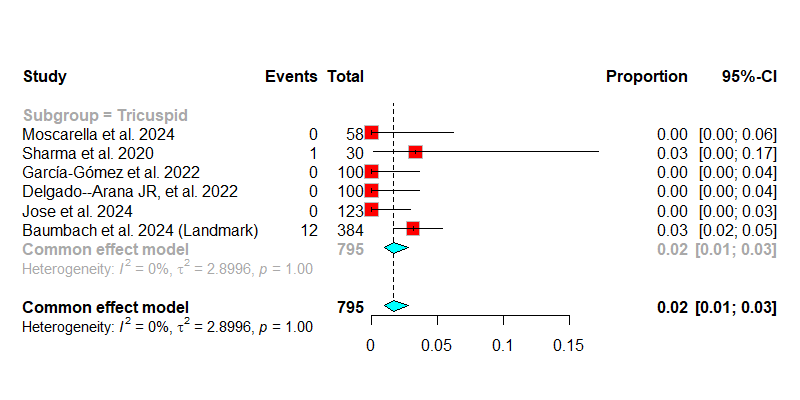
**

1. **Other cardiovascular rehospitalization**

**
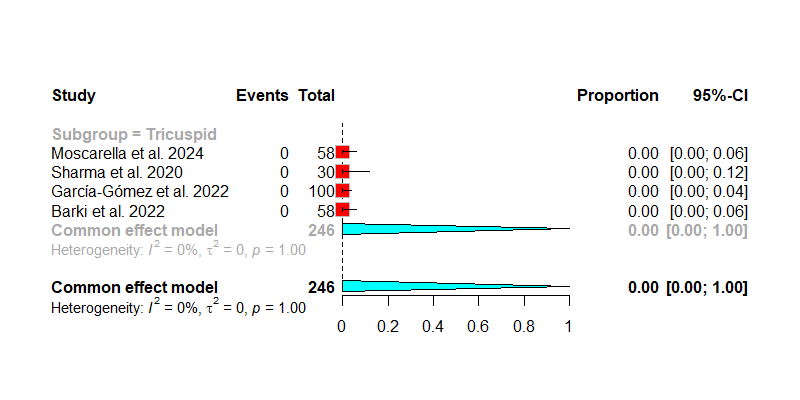
**

1. **Incidence of patients with mean aortic gradient > 20 mmHg**

**
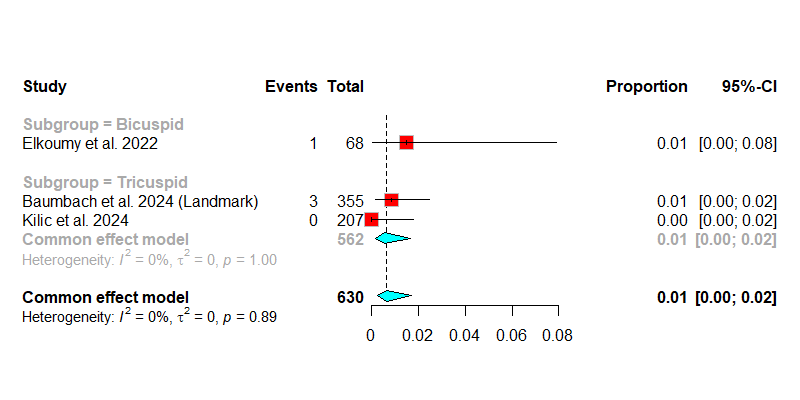
**

**Supplementary Figure 4:** Outcomes following Myval THV in patients with aortic stenosis and aortic diseases **one-year**: (A)All-cause mortality, (B) Cardiovascular mortality, (C) Non-cardiovascular mortality, (D) All stroke, (E) New permanent pacemaker implantation, (F) Acute kidney injury type stages 2,3 and 4 (G) Acute kidney injury, (H) Myocardial infarction, (I) Other cardiovascular rehospitalisation, (J) Non-cardiovascular rehospitalisation

1. **All causes mortality**


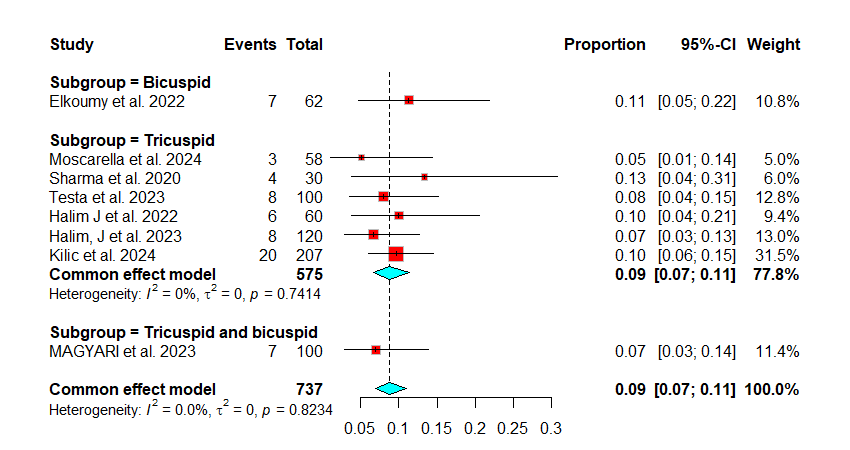


1. **Cardiovascular mortality**


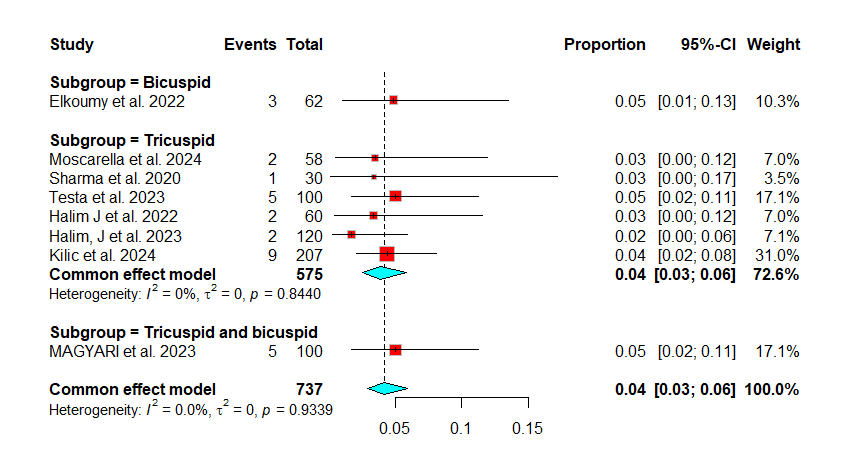


1. **Non cardiovascular mortality**


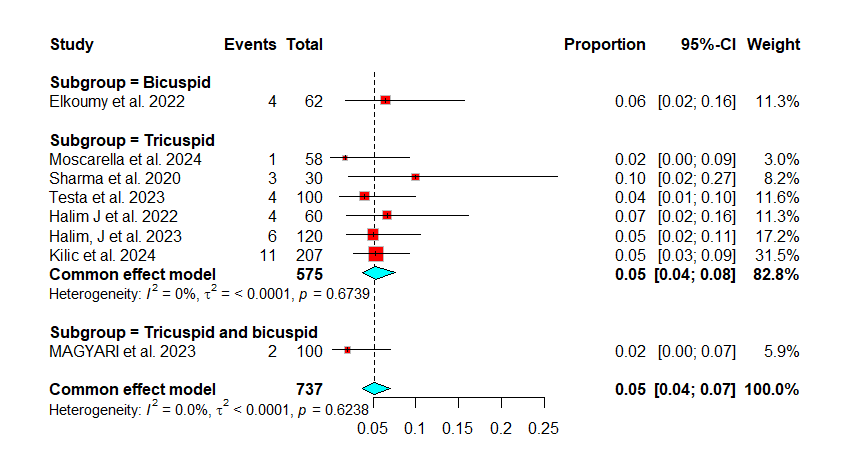


1. **All stroke**


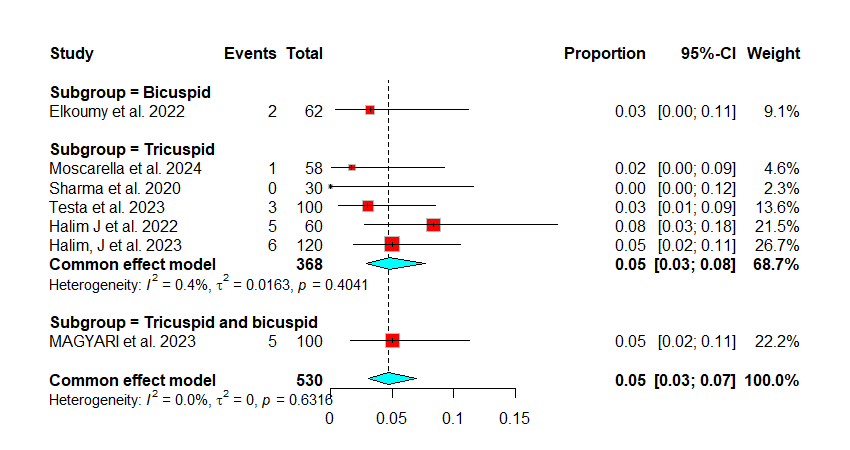


1. **New permanent pacemaker implantation**


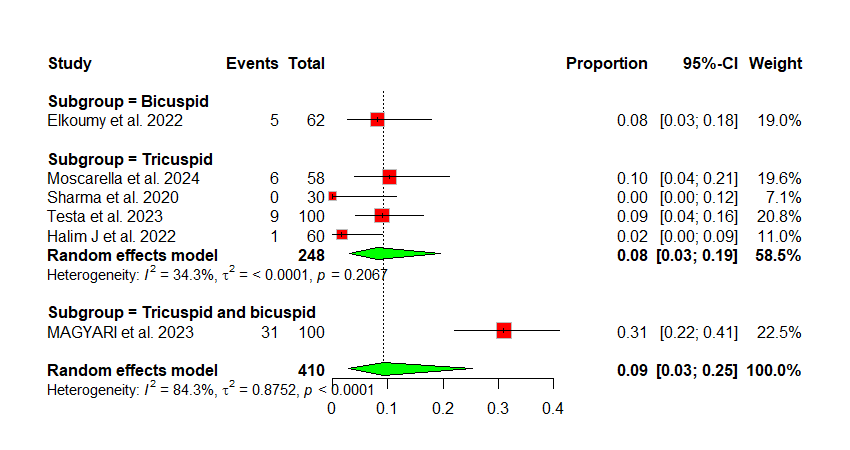


1. **Acute kidney injury type stages 2,3 and 4**


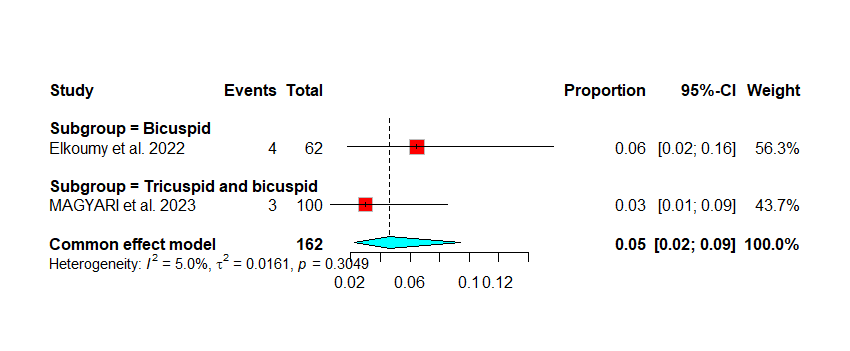


1. **Acute kidney injury**

**
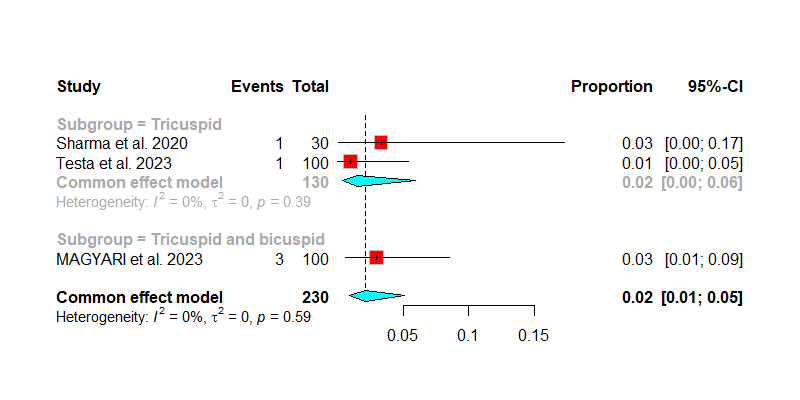
**

1. **Myocardial infarction**


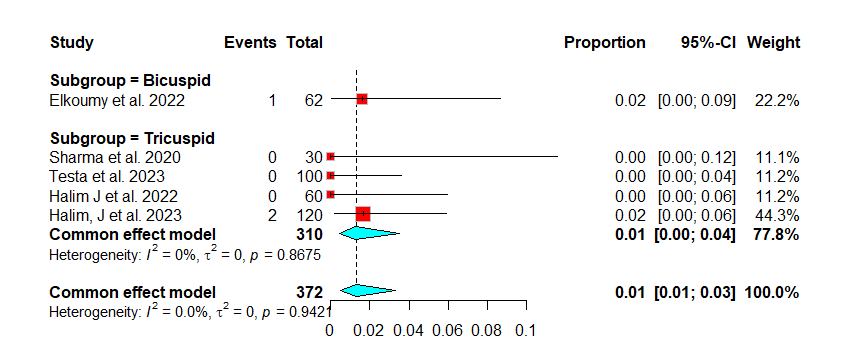


1. **Other cardiovascular rehospitalisation**


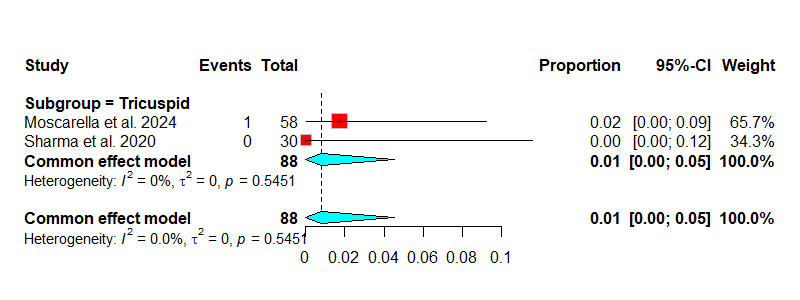


1. **Non-cardiovascular rehospitalisation**


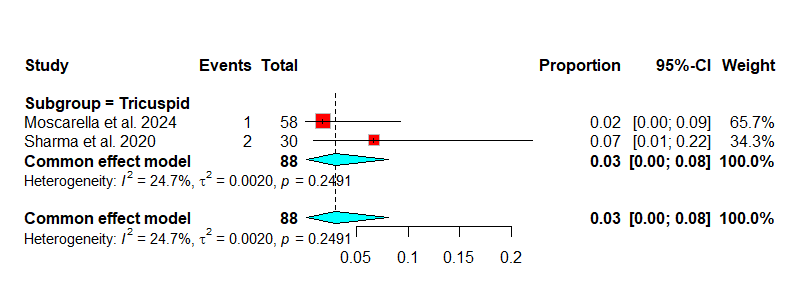


**Supplementary Figure 5:** Outcomes following Myval THV in patients with aortic stenosis and aortic diseases **two-year:** (A) All-cause mortality, (B) Cardiovascular mortality, (C) Non-cardiovascular mortality, (D) All stroke, (E) New permanent pacemaker implantation, (F) Acute kidney injury, (G) Myocardial infarction

1. **All causes mortality**

**
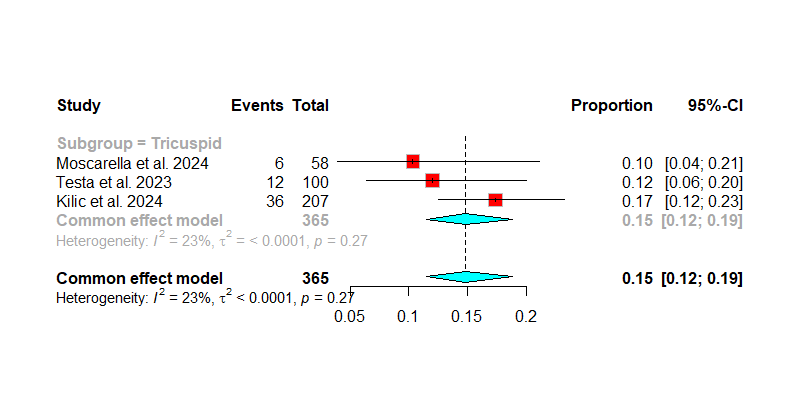
**

1. **Cardiovascular mortality**

**
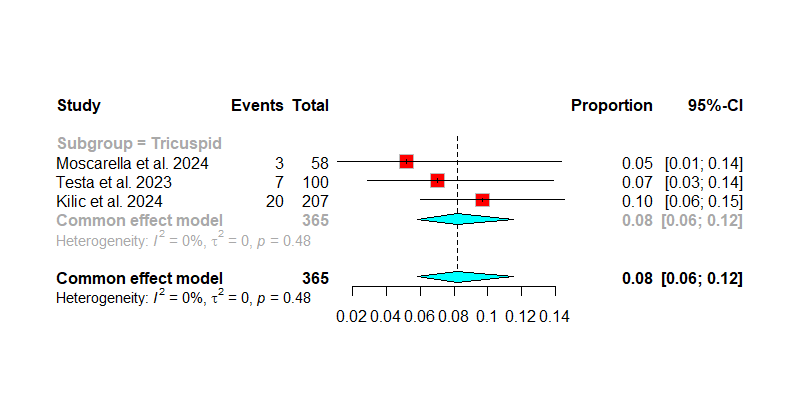
**

**(C) Non cardiovascular mortality**

**
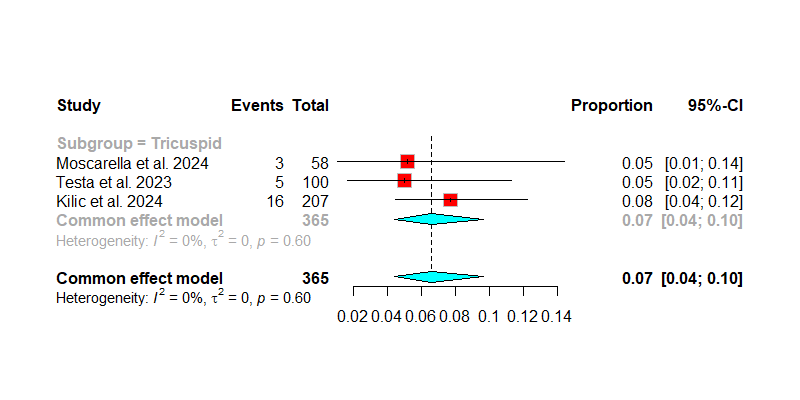
**

**(D) All stroke**

**
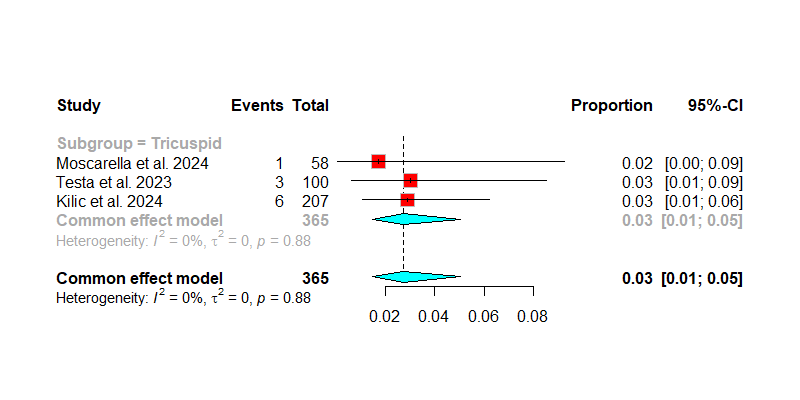
**

**(E) New permanent pacemaker implantation**

**
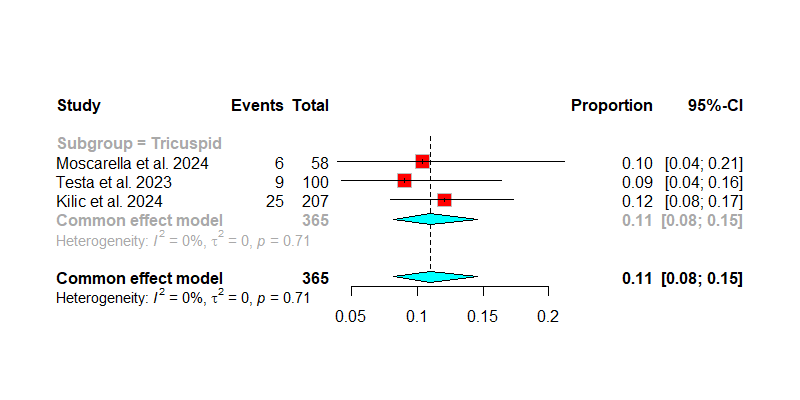
**

**(F) Acute kidney injury**


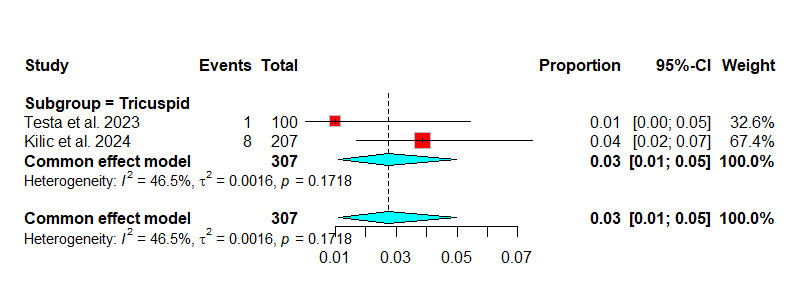


**(G) Myocardial infarction**


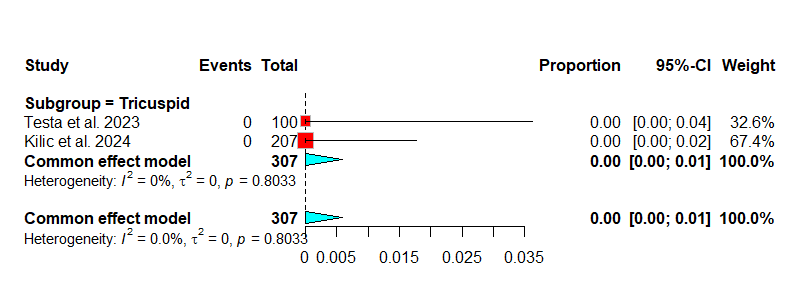

Supplement: Supplementary Data 1 [file mmc1.docx]
